# Global publication trends and research trends of necroptosis application in tumor: A bibliometric analysis

**DOI:** 10.3389/fphar.2023.1112484

**Published:** 2023-04-24

**Authors:** Yun-Yun Wu, Chang-chun Li, Xiao Lin, Feng Xu, Su-Kang Shan, Bei Guo, Fu-Xing-Zi Li, Ming-Hui Zheng, Qiu-Shuang Xu, Li-Min Lei, Jia-Yue Duan, Ke-Xin Tang, Ye-Chi Cao, Ling-Qing Yuan

**Affiliations:** ^1^ Department of Metabolism and Endocrinology, National Clinical Research Center for Metabolic Diseases, The Second Xiangya Hospital, Central South University, Changsha, China; ^2^ Department of Radiology, The Second Xiangya Hospital, Central South University, Changsha, China

**Keywords:** necroptosis, tumor, cancer, bibliometric study, VOSviewer, citespace

## Abstract

**Introduction:** Necroptosis is an alternative, caspase-independent programmed cell death that appears when apoptosis is inhibited. A gowing number of studies have reflected the link between necroptosis and tumors. However, only some systematical bibliometric analyses were focused on this field. In this study, we aimed to identify and visualize the cooperation between countries, institutions, authors, and journals through a bibliometric analysis to help understand the hotspot trends and emerging topics regarding necroptosis and cancer research.

**Methods:** The articles and reviews on necroptosis and cancer were obtained from the Web of Science Core Collection on 16 September 2022. Countries, institutions, authors, references, and keywords in this field were visually analyzed by CtieSpace 5.8.R3, VOSviewer 1.6.18, and R package “bibliometrix.”

**Results:** From 2006 to 2022, 2,216 qualified original articles and reviews on necroptosis in tumors were published in 685 academic journals by 13,009 authors in 789 institutions from 75 countries/regions. Publications focusing on necroptosis and cancer have increased violently in the past 16 years, while the citation number peaked around 2008–2011. Most publications were from China, while the United States maintained the dominant position as a “knowledge bridge” in necroptosis and cancer research; meanwhile, Ghent University and the Chinese Academy of Sciences were the most productive institutions. Moreover, only a tiny portion of the articles were multiple-country publications. Peter Vandenabeele had the most significant publications, while Alexei Degterev was most often co-cited. Peter Vandenabeele also gets the highest h-index and g-index in this research field. Cell Death and Disease was the journal with the most publications on necroptosis and cancer, which was confirmed to be the top core source by Bradford’s Law. At the same time, Cell was the leading co-cited journal, and the focus area of these papers was molecular, biology, and immunology. High-frequency keywords mainly contained those that are molecularly related (MLKL, NF-kB, TNF, RIPK3, RIPK1), pathological process related (necroptosis, apoptosis, cell-death, necrosis, autophagy), and mechanism related (activation, expression, mechanisms, and inhibition).

**Conclusion:** This study comprehensively overviews necroptosis and cancer research using bibliometric and visual methods. Research related to necroptosis and cancer is flourishing. Cooperation and communication between countries and institutions must be further strengthened. The information in our paper would provide valuable references for scholars focusing on necroptosis and cancer.

## 1 Introduction

Necroptosis is a programmed non-apoptotic pattern of cell death that shows remarkable similarities in the mechanism of apoptosis and morphology of necrosis (Christofferson and Yuan, 2010). Alexei Degterev initially proposed the term “necroptosis” in 2005, during which they found that the necroptosis pathway can be activated when intracellular apoptotic signaling is absent (Degterev et al., 2005). Receptor-interacting serine/threonine protein kinase 1 (RIKP1), RIPK3, and their substrate, mixed lineage kinase domain-like pseudokinase (MLKL), are recognized as key molecules in mediating this complicated process, and the translocation of phosphorylated MLKL is essential for disrupting cell membrane integrity during necroptosis pathway (Cai and Liu, 2014). Activation of death domain receptors [e.g., tumor necrosis factor receptor (TNFR) and Fas/FasL] and Toll-like receptors (TLR4 and TLR3) triggers necroptosis; then, they recruit the adapter proteins to interact with RIPK3. When caspase-8 is inactivated, RIPK1 recruits RIPK3 to form the RIPK1/RIP3 complex, which recruits and phosphorylates MLKL, forming necrosome within highly phosphorylated inositol phosphate (IP6). MLKL oligomers translocate to patches in plasma membrane rich in phosphatidylinositol phosphate (PIP) and form large pores, which results in the rupture of cellular membrane accompanied by uncontrollable delivery of intracellular substances, as well as the translucency of cytoplasm and swelling of organelles (Rosenbaum et al., 2009; Vandenabeele et al., 2010; Bertheloot et al., 2021). Therefore, as an alternative RIPK-dependent cell death mode, necroptosis may have vital implications for designing several research strategies in diverse areas. Additional understanding is needed to explore the full potential of necroptosis.

There is compelling evidence that necroptosis plays an integral role in tumorigenesis and cancer progression and participates in infection, sterile inflammation, myocardial infarction, and neurodegenerative diseases (Weng et al., 2014; Ofengeim et al., 2015; Seehawer et al., 2018; Qin et al., 2019). Among them, researches focusing on necroptosis and cancer are worthy of special attention since the age of onset is gradually becoming younger and younger with increasing incidence. How to fight against cancer has become one of the top priorities for medical research worldwide. Even though significant efforts have been devoted to cancer research, some anticancer therapies may cause side effects due to drug resistance. Therefore, there is an urgent requirement to develop new and effective treatments.

Thanks to its immense potential, necroptosis has drawn attention in the cancer field. Several extraordinary articles on necroptosis and tumors from multifarious aspects have recently been published. One of the hotspots for this is anti-tumor immunity. Current anti-cancer drugs induce apoptosis in cancer cells, and anti-apoptotic mutations in cancer cells are the leading cause of drug resistance. However, targeting cell death mechanisms other than apoptosis may present a new avenue for cancer treatment. Studies have shown that inducing cancer cells to undergo necroptosis may play a role in killing tumors. In a recent study, researchers from the University of Washington and Rutgers University found that injecting necroptotic cells into mouse tumors directed killer T cells to attack malignant tumors and slow their growth (Snyder et al., 2019). The researchers also found that allowing tumor cells to express an enzyme that induces necroptosis was sufficient to initiate the process of tumor shrinkage. This strategy could improve the efficacy of existing immunotherapies. Furthermore, scientists from VIB-UGent Center for Medical Biotechnology have made significant progress by inducing the production of MLKL protein in cancer cells, promoting necroptosis in tumor cells, and triggering recognition by the immune system (van Hoecke et al., 2018). Interestingly, the MLKL protein also induces the immune system to attack surviving tumor cells while mediating explicitly programmed cell death without affecting the apoptotic process. Such treatment inhibits primary tumor growth in mice and prevents the spread of untreated tumors. There is a new and exciting approach to fighting tumors in addition to the mainstream induction of necroptosis in cancer cells. In 2016, Tania Løve Aaes et al. confirmed that necroptosis in tumors is a kind of immunogenic cell death for the first time (Aaes et al., 2016). They injected the vaccine made with necroptotic cancer cells, and the result is that the vaccination with the necroptotic cancer cells can inhibit tumor growth in mice. This research indicated that necroptotic tumor cells were immunogenic, which could activate efficient anti-tumor immunity in the body. This demonstrates that necroptosis has significant potential for anti-tumor immunity. Despite intensive research on necroptosis and tumors, there is no comprehensive and rigorous evidence on the publication trends, distribution of countries/regions, authors, institutions, journals, and their cooperation in this research field until now.

Several systemic reviews have been published in recent years, and bibliometrics is one of the most efficient and popular approaches (Mulet-Forteza et al., 2021). Bibliometrics was first proposed in 1969 as a discipline that encourages mathematical and statistical judgment. Correspondingly to the attributes of the literature database, bibliometrics can not only help researchers to clarify indicators by analyzing the evolution and dynamics of qualitative and quantitative information in a particular field but also grasp future research trends through evaluation and overview of the contribution of countries/regions, institutions, authors, and journals (Chen and Song, 2019; Vanzetto and Thomé, 2019; Colares et al., 2020; Ma et al., 2020). Moreover, some unique indicators are essential in bibliometric analysis. Initially developed for the quantitative study of science, bibliometric indicators have two divisions. The first division can be extracted directly from the bibliographic database, including publication trends, citation counts, and citation analysis. And the second division can be obtained by mathematically manipulating data from the bibliographic database, such as H-index, G-index, etc. This study is the first bibliometrics analysis related to necroptosis and tumors, which will be extremely useful for this field. Therefore, bibliometrics is befitting for laying a foundation in the research field related to necroptosis and cancer.

We undertook this study to offer insights into research spots and therapeutic cancer targets related to necroptosis from 2006 to 2022 with CiteSpace and VOSviewer software. The study objectively describes the scientific domain from four aspects as follows.

(1) We quantify the volume of the annual research products related to necroptosis and cancer in the central databases (i.e., Web of Science) from 1 January 2006 to 16 September 2022.
(2) We identify the principal authors, journals, countries/regions, and institutions publishing research on necroptosis and tumors and analyze the most co-cited authors to elaborate on the knowledge base of necroptosis through CiteSpace and VOSviewer.
(3) We present the reference spectroscopy, word dynamics, trend topics, thematic evolution, factorial analysis, and some other data analyzed by R packages “bibliometrix” and its online platform “bibioshiny.”
(4) Last but not least, exploring the knowledge framework and hotspots evolution and advancing an agenda for emerging topics related to necroptosis and tumors by conducting keywords analysis and co-cited reference burst analysis. In a word, these three objectives cover the *status quo* and the latest trends in necroptosis research.

## 2 Materials and methods

### 2.1 Data collection

**FIGURE 1:**
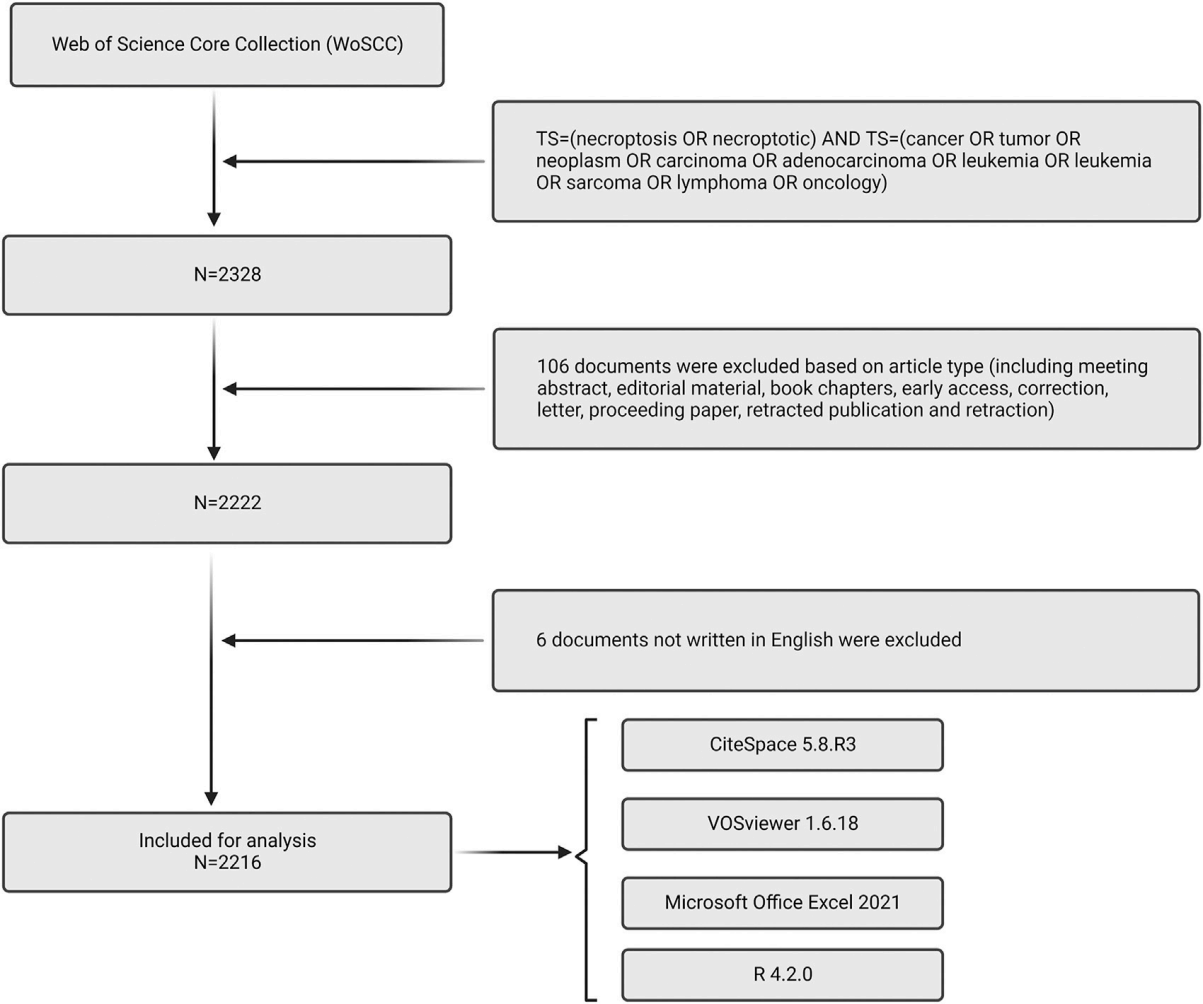
Flow chart of data collection in this study.

Web of Science Core Collection (WoSCC), an influential database generally used in the bibliometric analysis, furnishes statistical evidence for this study. We downloaded the information within 1 day on 16 September 2022. The query equation was set as follows: TS = (necroptosis OR necroptotic) AND TS = (cancer OR tumor OR neoplasm OR carcinoma OR adenocarcinoma OR leukemia OR leukemia OR sarcoma OR lymphoma OR oncology), and the period of the search were limited from 1 January 2006 to 16 September 2022. 2,323 documents were retrieved by using this strategy to search the database. Of these, 106 papers were excluded due to irrelevant article types, including meeting abstracts, editorial materials, early access, book chapter, letters, retractions, proceedings papers, new items, etc. Moreover, 6 documents were written in non-English and thus excluded for further research. This manual screening process narrowed the collection to 2,211 documents, including 1,698 articles and 518 reviews. Search results were carried out in the format of “Plain Text” and stored in the format of “download.txt” since CiteSpace could only read files in this form (Figure 1).

### 2.2 Data analysis and visualization

All of the available data were yielded in Web of Science Core Collection and imported to Microsoft Excel Office 2021 (Microsoft, Redmond, Washington, United States), R (4.2.0), VOSviewer (1.6.18), and CiteSpace 5.8.R3, for further visual analysis.

VOS viewer, a program serving for creating, visualizing, and exploring bibliometric maps, is one of the essential implements to build authors, countries, and keyword maps based on collaborative data or co-occurrence data, respectively (van Eck and Waltman, 2010). The program equips a viewer to achieve a comprehensive and objective review of bibliometric maps, and the graphic representation of the VOS viewer provides intuitive interpretation and understanding. We used VOS viewer 1.6.18 in this present study for building network visualization of keywords density, co-cited authors, journals, countries, and citations, as well as overlay visualization based on bibliographic data. We choose the full-counting method and set thresholds shown in the corresponding chapter. In the cluster map, node size represents frequency, colors represent related clusters, the link line reflects co-occurrence relationship, and the thickness of the link line reflects cooperation degree, depending on the publications two researchers co-authored, or two keywords co-occurred.

CiteSpace is a citation visualization analysis tool that does well in exploring current cooperation, key points, internal framework, potential hot spots, and evolution in a particular field (Chen, 2004). Hence, we use CiteSpace 5.8.R3 to analyze the co-occurrence of countries/regions and institutions, keywords timeline, keywords bursts, citation bursts, and dual-map of journals. The settings were set as follows: timespan (2006–2022), years per slice (Christofferson and Yuan, 2010), pruning (pathfinder/pruning sliced networks), selection criteria (Top N = 50), the minimum duration of burstness (2 years), cluster labels were extracted by light semantic indexing (LSI) and the log-likelihood ratio (LLR) algorithm, and others followed the default. In CiteSpace visualization, node size represents frequency, and the link line reflects the co-occurrence relationship. The colors of nodes and lines represent different years, which vary from purple to red from 2006 to 2022. Nodes with purple circles indicate a high betweenness centrality (≥ 0.10), and they were considered as a bridge between different networks (Chen, 2004; Chen et al., 2012; Chen, 2017).

Bibliometrix is a comprehensive R language package for bibliometric analysis and scientific visualization, developed and maintained by Massimo Aria and Corrado Cuccurullo (https://www.bibliometrix.org/index.html). Bibliometrix is able to transform the format of the data downloaded from the SCOPUS, Web of Science, Cochrane Database of Systematic Reviews (CDSR), and RISmed PubMed/MedLine databases so that they can be used in an R environment. Bibliometrix includes two prominent families of functions that cover the analytical techniques involved in bibliometrics: 1) the extraction of fundamental bibliometric analysis and analytical metrics; and 2) the mining of conceptual, intellectual and social structures related to the literature.

To accurately analyze and visualize the trend of published documents number, we used Microsoft Office Excel 2021 and R 4.2.0 to complete this work. Besides, we gained the 2019 impact factor (IF) and Journal citation reports (JCR) division of journals from the Web of Science InCites Journal Citation Reports on 30 September 2022.

## 3 Results

### 3.1 The trends of annual publication outputs

**FIGURE 2:**
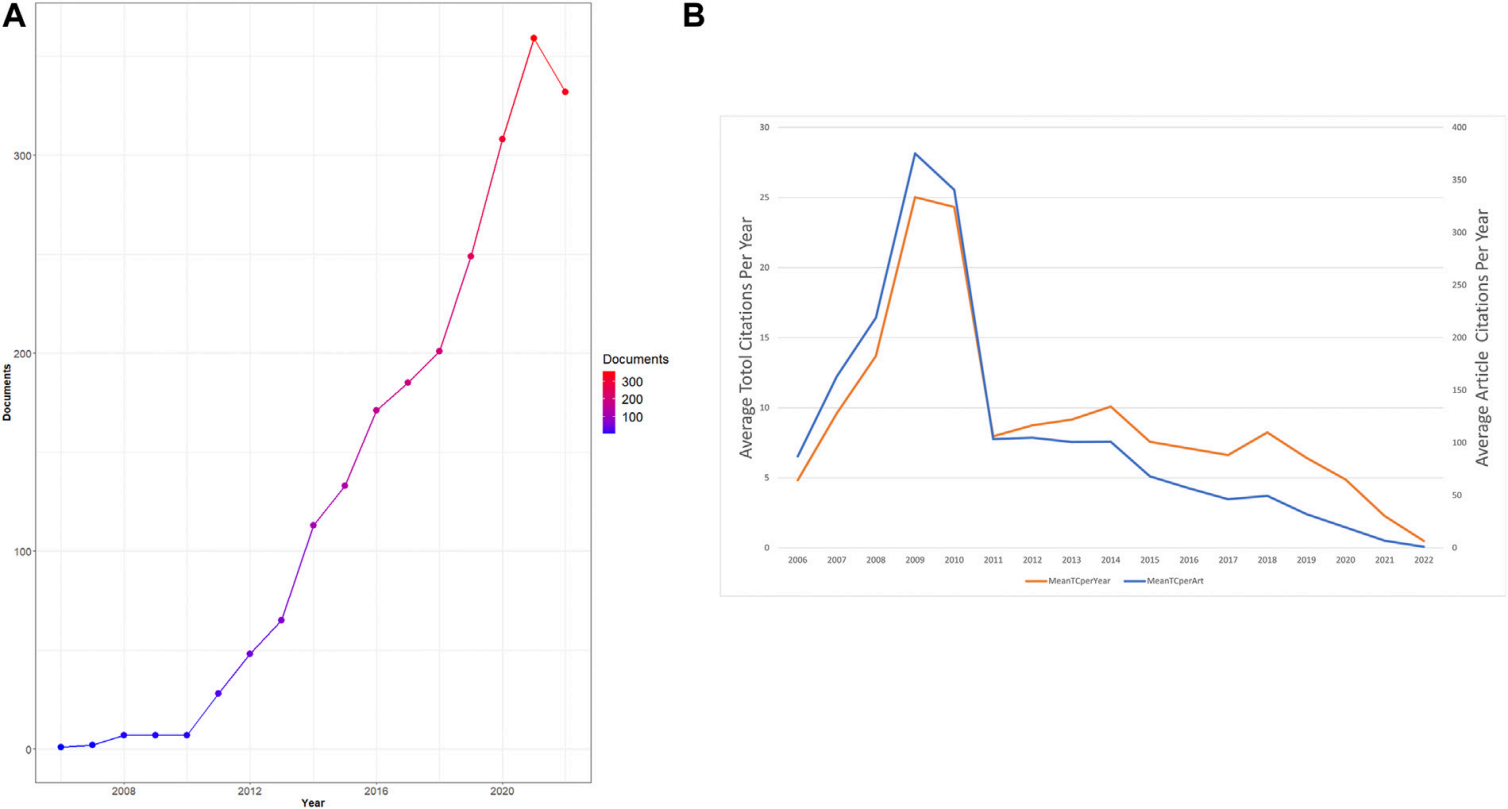
Analysis of total publications. **(A)**Trends of necroptosis publication from 2006 to 2021. **(B)** Trends of average total/article citations per year.

It has been recognized that the development landscape in a research field closely correlates with the volume of articles published in each period. As the data collection section described, we collected 2,216 eligible documents published between 2006 and 2022. As shown in Figure 2A, the number of articles related to necroptosis and cancer has steadily increased over time. The global output of this research field remained low from 2006 to 2010, and it started to rise stably between 2006 and 2022, reflecting that interest in necroptosis research has burgeoned in the last 11 years. Moreover, the average article/total citation per year is shown in Figure 2B. The citation number per year peaked around 2008–2011, which suggests that the most influential articles in this field were published during these years.

### 3.2 Distribution of countries/regions and institutions

**FIGURE 3:**
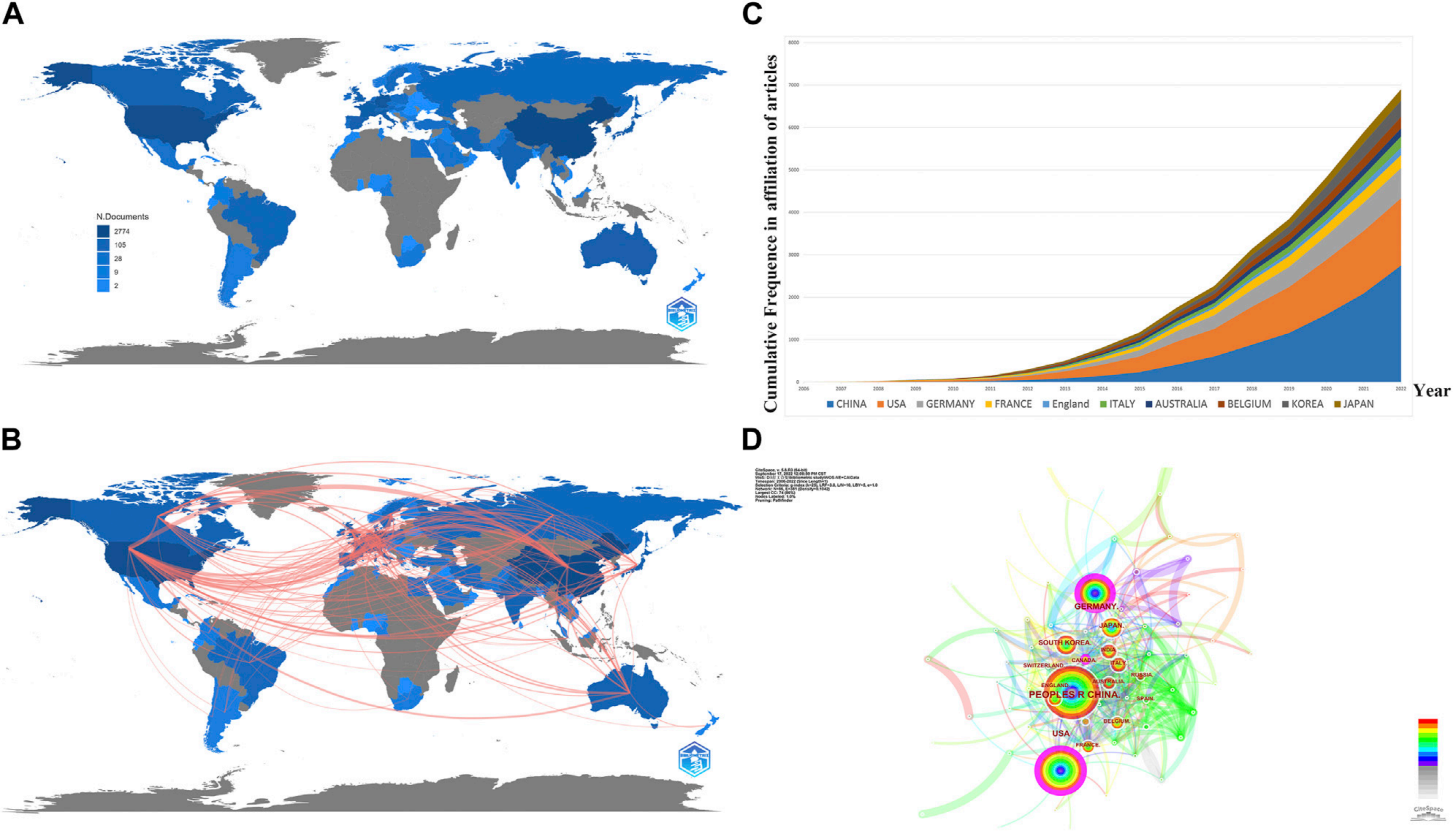
Analysis of publications from different countries/regions. **(A)** Geographic distribution map based on the total publications of different countries/regions. **(B)** Countries’ geographic collaboration network. **(C)** Top 10 productive countries’ frequencies in all publications over time. **(D)** CiteSpace network visualization map of countries/regions of the articles related to necroptosis in cancer research.

In order to explore the publications among different countries/regions, a geographical collaboration analysis was performed (Figures 3A, B). A total of 2,216 documents (including 1,698 articles and 518 reviews) were published from 75 countries and 789 institutions (Table 1). As is shown in Figure 3A, China.

**TABLE 1:** Top 10 productive countries/regions and institutions related to necroptosis in cancer research.

Rank	Countries/Regions	Count	Centrality	Institution	Countries/Regions	Count	Centrality
1	China	861	0.12	Univ Ghent	Belgium	76	0.12
2	United States	553	0.29	Chinese Acad Sci	China	69	0.09
3	Germany	253	0.22	Flanders Inst Biotechnol VIB	Belgium	66	0.03
4	Korea	130	0.08	Central South Univ	China	59	0.04
5	Japan	103	0.05	Inst National de la Sante et de la Recherche Medicale (INSERM)	France	53	0.08
6	Belgium	85	0.03	Harvard Univ	United States	52	0.12
7	Italy	84	0.03	Univ of Texas System	United States	49	0.01
8	England	82	0.10	Zhejiang Univ	China	46	0.05
9	France	71	0.05	Udice French Research Univ	France	44	0.02
10	Australia	70	0.05	Sun Yat-sen Univ	China	43	0.04

(861, 38.85%) accounted for the most significant section of publications, with the United States (553, 24.96%) a close second, followed by Germany (253, 11.42%), and South Korea.

(130, 5.87%) Figures 3B, D are both collaborating visualization maps of countries. In Figure 3D different nodes represent different countries; the dimension of the node indicates the academic output from the corresponding country, and the surrounding color refers to the publications in corresponding years, which vary from 2006 to 2022. Moreover, the color of the links refers to the year the two countries first collaborated, and the thickness of the links indicates the number of their collaborated publications. According to Figure 3D, China, the United States, and Germany kept higher academic outputs over the past 16 years. Moreover, the dense links imply a booming landscape of dynamic cooperative behavior among different countries/regions. However, the outer circle of the node China is not presented with purple since the centrality was less than 0.1; only United States (centrality = 0.29), Germany (centrality = 0.22), and Canada (centrality = 0.12) are presented with a purple circle, indicates they act as a “bridge” node in the research field related to necroptosis and cancer. As for Figure 3B, we exhibited the top 5 countries’ production results over time. Obviously, we can see an increasing trend of publications by year, and China’s output grows fastest among them.

**FIGURE 4:**
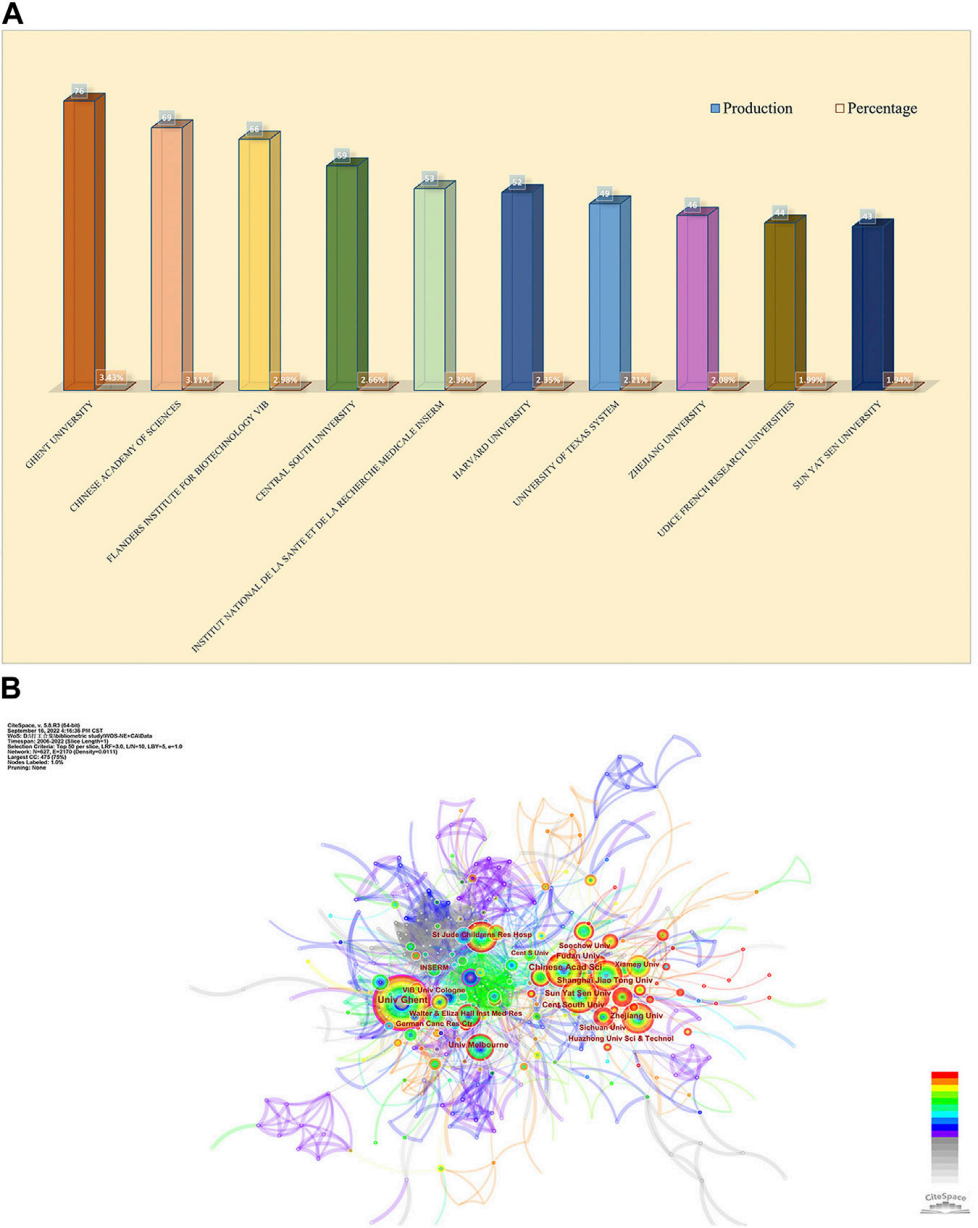
Analysis of publications from different institutions. **(A)** The chart of counts and percentages of the top productive 10 institutions. **(B)** CiteSpace network visualization map of institutions of the articles related to necroptosis in cancer research.

The design and principle are the same in Figure 4. The top 10 institutions were from China (3/10), the United States (2/10), Belgium (2/10), and France (2/10) (Figure 4A). The University of Ghent (76, 3.43%) published the most papers, followed by the Chinese Academy of Sciences (69, 3.11%), Flanders Institute for Biotechnology (66, 2.98%), Central South University (59, 2.66%), and Institut National de la Sante et de la Recherche Medicale (53, 2.39%) (Figure 4A). Besides, we can see from Figure 4B that there was active cooperation among them. Ghent University is the most productive institution, and its centrality is also larger than 0.10 (*n* = 76, centrality = 0.12), which implies that Ghent University occupies a key position in this field. By contrast, the Chinese Academy of Sciences (centrality = 0.09), Zhejiang University (centrality = 0.05), and Sun Yat-Sen University (centrality = 0.05) had a low centrality.

### 3.3 Authors and co-cited authors

**FIGURE 5:**
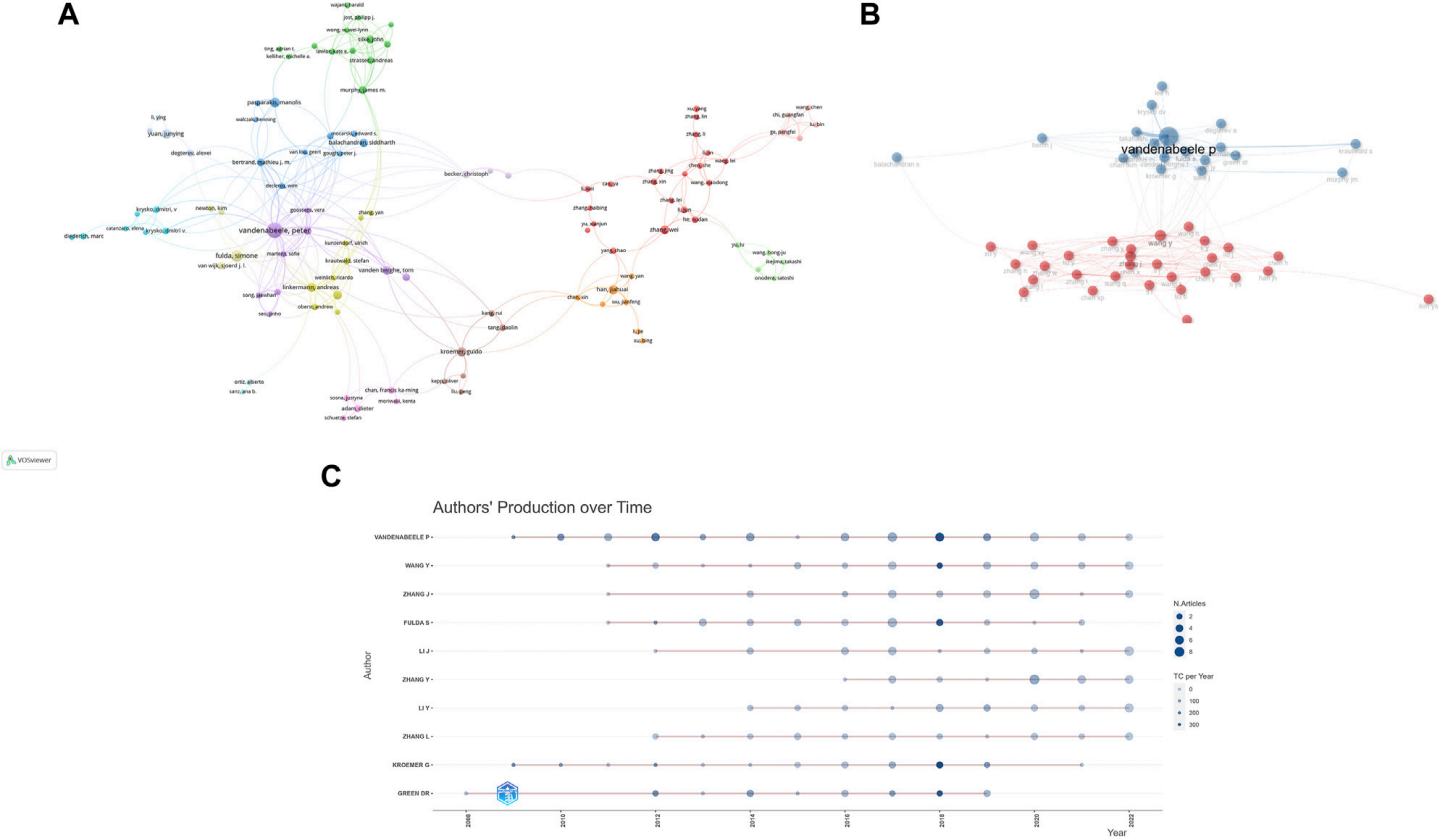
Analysis of several productive authors and co-cited authors. **(A)** The co-occurrence visualization map of author co-authorship analysis generated by VOSviewer. **(B)** Authors’ collaboration network performed by R tool “biblioshiny”. **(C)** Top 10 productive authors’ production over time.

In order to let people know the authors that contributed significantly to this field, the bibliometric analysis of authors and co-cited authors was performed. A total of 13,009 authors have been part of the work, actively investigating the underlying mechanisms of necroptosis and publishing papers. Among them, 169 authors had more than 5 publications in this field. We can see from Table 2 that Vandenabeele Peter (*n* = 42) accounts for the vast majority of publications, followed by Fulda Simone (*n* = 24) and Junying Yuan (*n* = 16). Vandenabeele Pete has the highest value in total link strength, followed by Takahashi Nozomi, Betrend Mathieu j.m, and Kroemer, guido, which suggested that these authors above had substantial leverage on each other’s production along with works from other scholars. In Figure 5A, the principle is that various nodes symbolize different authors, and the lines represent their cooperation. Moreover, the color of the nodes indicates that there are 11 clusters in academic collaboration. Figure 5B shows the result of a cluster analysis of the author collaboration network by the “bibliometrix” package. The clustering results compute 2 clusters, and geographical factors clearly influence collaboration between authors. The closest association is between Vandenabeele *p* and Takahashi Nozomi, Betrend Mathieu j.m, etc. “Co-cited authors” refers to at least two authors’ papers that were cited simultaneously so that these two or more authors form a co-cited relationship. Among 875 co-cited authors, 9 have been cited more than 500 times (Table 2). Degterev A (*n* = 1,082) was the author with the most significant number of cited papers, followed by Galluzzi L (*n* = 967). Figure 5C shows the most active author working on necroptosis and cancer during 2006–2022. We can see that 2018 was the most cited year for the output of influential authors in the field, and the publications from Vandenabeele *p*, Wang Y, Fulda S, Kroemer G, and Green DR were all cited over 300 times in 2018.

**TABLE 2:** The top 10 authors and co-cited authors of necroptosis in cancer research.

Rank	Author	Documents	Citations	Total link strength	Co-cited author	Citations	Total link strength
1	Vandenabeele, Peterb	42	5,831	82	Degterev, A	1,082	45,552
2	Fulda, Simone	24	752	18	Galluzzi, L	967	50,534
3	Junying, Yuan	16	2,740	14	He,sd	659	30,758
4	Pasparakis, Manolis	16	1,059	12	Vandenabeele, *p*	608	22,088
5	Kroemer, Guido	15	3,199	29	Newton, K	607	34,937
6	JiaHuai, Han	15	2018	20	Linkermann, A	549	25,801
7	Linkermann, Andreas	14	2,120	25	Sun, Lm	547	23,343
8	Vanden Berghe, Tom	13	3,359	26	Cho, Y	541	24,702
9	Green, Douglas r	13	2,761	13	Kaiser,wj	533	34,967
10	Wei, Zhang	13	180	7	Dongdelinger, Y	497	33,915

### 3.4 Journals and co-cited academic journals

We used VOSviewer to carry out co-citation and co-cited journal analysis, detecting the most promising and influential journals in the necroptosis and cancer field. The results showed that 2,216 articles associated with necroptosis and cancer were published in 685 academic journals (Table 3). The journal *Cell death and disease* (*n* = 106) occupied the vast majority of outputs, followed by *Cell death and differentiation* (*n* = 62) and the *International journal of molecular sciences* (*n* = 52). *Cell death and differentiation* (IF = 12.007) has the highest Impact factor (IF) among the top 10 journals. Moreover, it can be figured out from Table 2 that 70% of the top 10 journals belong to the Q1 JCR division. The potential influence of journals mainly depended on the counts of co-citations, indicating the journal’s impacts in a specific research field. Within the top 10 co-cited academic journals, five journals have citations over 4,000 times. As shown in Table 3, the journal with the highest number of co-citations is *Cell* (*n* = 6,763), followed by *Nature* (*n* = 6,083)*, Cell death, and differentiation* (*n* = 4,971). Based on the 2021 Journal citation reports (JCR), apart from the *Journal of biological chemistry* and *Cancer Research*, nearly all the co-cited journals were located in the Q1 JCR region.

**TABLE 3:** Top 10 journals and co-cited journals related to necroptosis in cancer research.

Rank	Journal	Counts	If (2021)	JCR (2021)	Co-cited journal	Citation	If (2021)	JCR (2021)
1	Cell death and disease	106	9.705	Q1	Cell	6,763	66.850	Q1
2	Cell death and differentiation	62	12.077	Q1	Nature	6,083	69.504	Q1
3	International journal of molecular sciences	52	6.208	Q1	Cell death and differentiation	4,971	12.077	Q1
4	Cancers	45	6.575	Q2	Journal of biological chemistry	4,433	5.486	Q2
5	Scientific reports	44	4.996	Q1	Proc Natl Acad Sci United States	4,078	10.700	Q1
6	Oncotarget	36	5.168	Q1	Science	2,885	63.798	Q1
7	Frontiers in immunology	32	8.786	Q1	Cell death and disease	2,870	9.705	Q1
8	Cancer letters	30	9.756	Q1	Molecular Cell	2,817	19.328	Q1
9	Frontiers in Oncology	29	5.738	Q2	Immunity	2,204	43.474	Q1
10	Plos one	27	3.752	Q2	Cancer Research	2,187	13.312	Q2

#Proc Natl Acad Sci United States (Proceedings of the national academy of sciences of the United States of America); IF, impact factor; JCR, journal citation reports.

**FIGURE 6:**
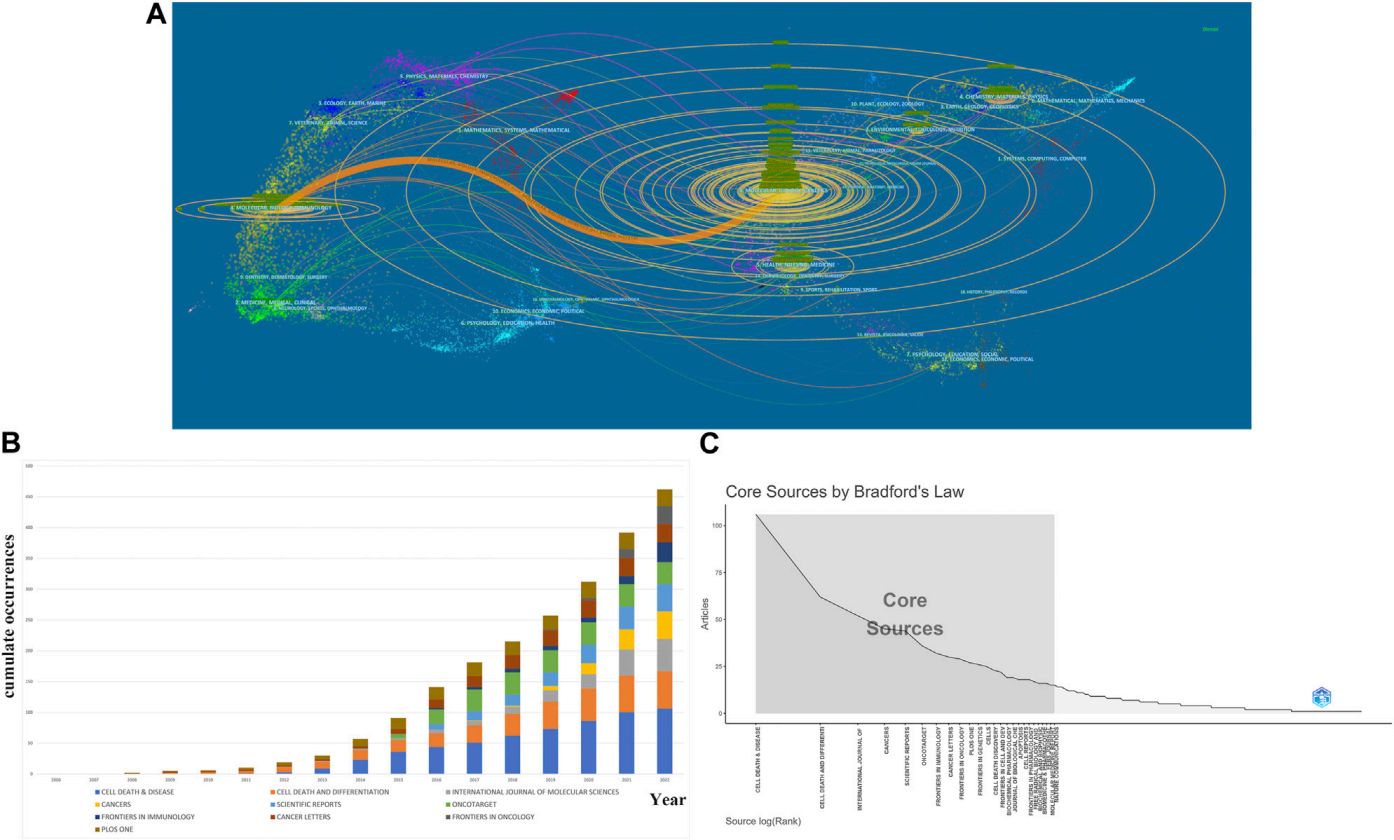
Analysis of journals and co-cited journals. **(A)**A dual-map overlap of journals related to necroptosis in cancer research carried out by CiteSpace. **(B)** Top 10 productive journal publications over time. **(C)** The grey box refers to core journals in this field.

Figure 6A is the journal’s dual-map overlay, indicating the journal topic distribution. The left part of the map was the citing journals, while the right part was the cited journals. The labels of different color clusters represent the discipline of the corresponding journals. The thickest path from left to right is the cited line, which reflects the citation’s ins and outs. The horizontal axis of the ellipse is positively correlated to the number of authors, while the longitudinal axis is positively correlated to the publications of journals. As shown in Figure 6A, an orange path suggests that the studies from the Molecular/Biology/Genetics journals were mostly cited from studies in Medical/Biology/Immunology journals. Moreover, Figure 6B shows the high-impact journals’ production over time, and Figure 6C shows the core journals calculated by Bradford’s Law.

### 3.5 Co-cited references and references burst

In this section, we analyzed the co-cited references and references burst, identifying landmark papers and theme evolution in this field. Firstly, we used VOS viewer to conduct the analysis of the co-cited references. As a research approach to estimate the relationship between articles, co-citation is defined as two or more articles being cited by one another simultaneously, and these two articles are recognized as co-citation references. Table 4 shows the top 10 frequently co-cited references among the 86,456 cited references retrieved, 7 of which were cited more than 400 times. Among them, the paper entitled “Mixed lineage kinase domain-like protein mediates necrosis signaling downstream of RIP3 kinase” (Sun et al., 2012), published in *Cell* by Liming Sun et al., in 2012, had the most citations (*n* = 529), immediately followed by an article named “Chemical inhibitor of non-apoptotic cell death with therapeutic potential for ischemic brain injury” (Degterev et al., 2005) (*n* = 509) published in *Cell* in 2005. Moreover, six of the top 10 co-cited references were published in *Cell, Nature, and Science*.

**TABLE 4:** Top 10 co-cited references related to necroptosis in cancer research.

References	Journal	Publication year	Citations	Type
Mixed lineage kinase domain-like protein mediates necrosis signaling downstream of RIP3 kinase	Cell	2012	529	Article
Phosphorylation-driven assembly of the RIP1-RIP3 complex regulates programmed necrosis and virus-induced inflammation	Cell	2009	509	Article
Chemical inhibitor of non-apoptotic cell death with therapeutic potential for ischemic brain injury	Nat Chem Biol	2005	499	Article
Receptor interacting protein kinase-3 determines cellular necrotic response to TNF-alpha	Cell	2009	486	Article
Molecular mechanisms of necroptosis: an ordered cellular explosion	Nature reviews	2010	434	Reviews
RIP3, an energy metabolism regulator that switches TNF-induced cell death from apoptosis to necrosis	Science	2009	421	Article
Identification of RIP1 kinase as a specific cellular target of necrostatins	Nat Chem Biol	2008	418	Article
Mixed lineage kinase domain-like protein MLKL causes necrotic membrane disruption upon phosphorylation by RIP3	Mol cell	2014	321	Article
Necroptosis and its role in inflammation	Nature	2015	305	Review
Fas triggers an alternative, caspase-8-independent cell death pathway using the kinase RIP as effector molecule	Nature immunology	2000	285	Article

**FIGURE 7:**
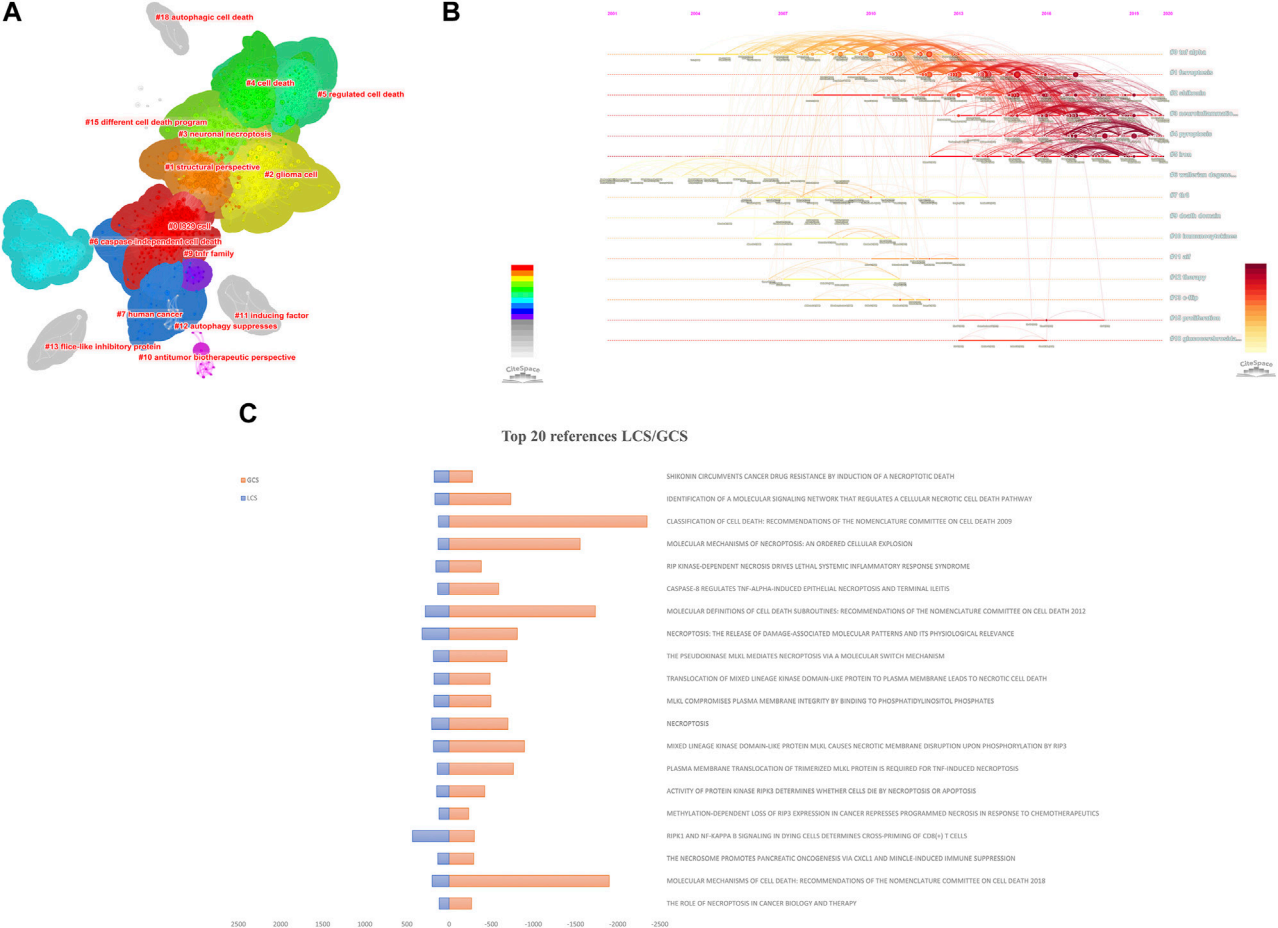
Analysis of co-cited references. The knowledge map **(A)** and the timeline view **(B)** of references related to necroptosis in cancer research. **(C)** The Top 20 references with the highest LCS/GCS.

Citespace is a reliable tool for constructing a network of co-cited references. As shown in Figure 7A, there are 15 clusters in the knowledge map. Modularity Q (0.717) and Mean Silhouette (0.8846) values were more significant than 0.5, indicating that the statistical significance of groups and credibility were convincing. The first cluster label on the knowledge map was “#0 tnf alpha,” and the second cluster label was “#1ferroptosis”.

Simultaneously, we construct the references timeline view to visualize the evolution of research hotspots from 2006 to 2022 (Figure 7B). Nodes with warm-tone colors imply that the references were cited in recent years. Cluster “#2 shikonin”, “#3 neuroinflammation”, “#4 pyroptosis”, and “#5 iron” are still hotspots for necroptosis and cancer research now.

Local citation score (LCS) is the number of citations of the article in the local (all downloaded literature), which indicates the number of citations of authors in the small field of study and is the metric that deserves our most attention when conducting a literature analysis. Global citation score (GCS) is the total number of citations for the article in the WOS database. The LCS is a subset of the GCS, the metric we usually use when searching by the number of citations. A paper with a high GCS and a low LCS will likely be more influential in other areas. Figure 7C shows this field’s top 20 references with high LCS/GCS.

**FIGURE 8:**
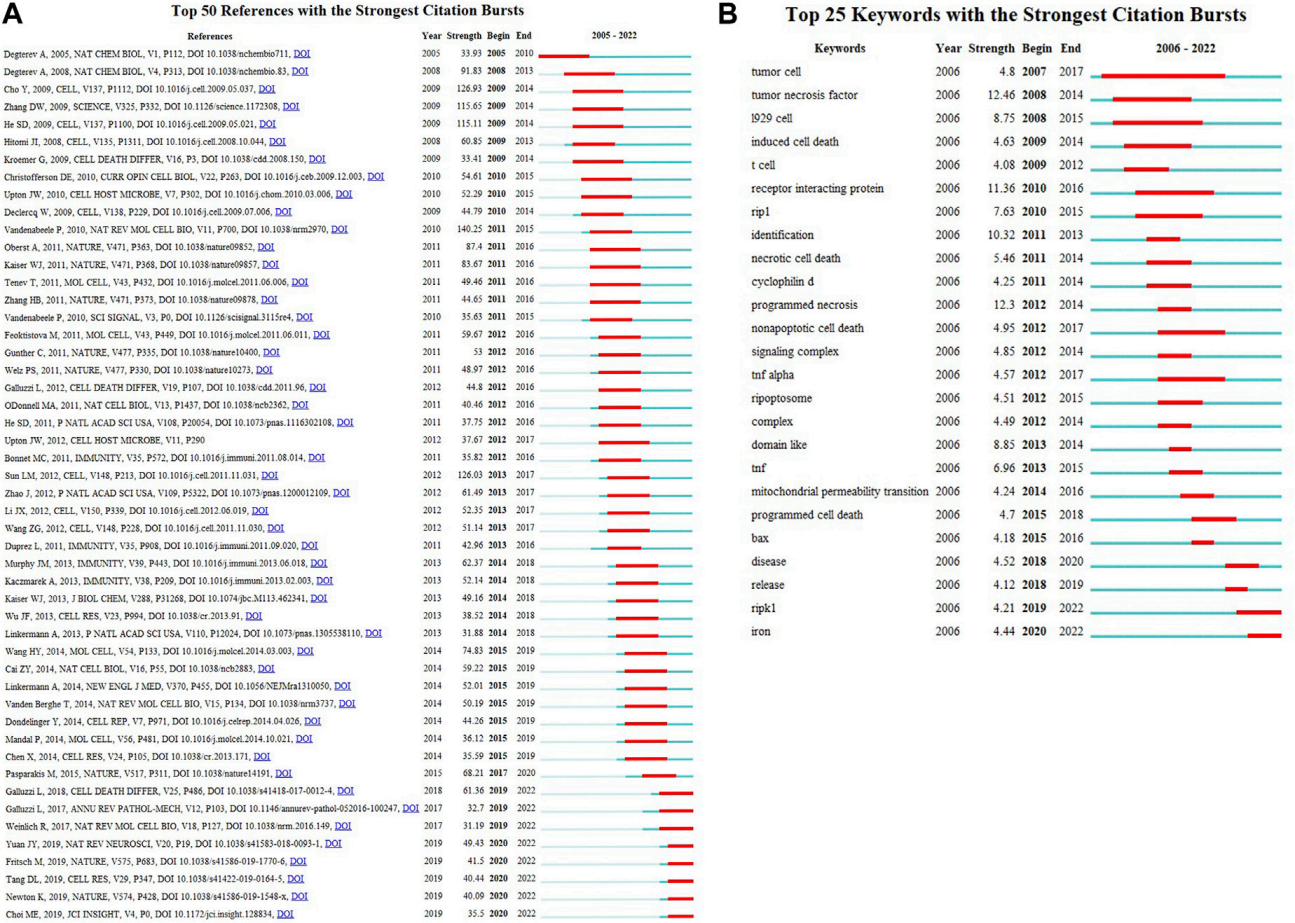
Top 50 **(A)** references and Top 25 **(B)** keywords with the strongest citation bursts.

Based on references cited frequency in a certain period, we set at least 2 years of burst duration in CiteSpace and detected 25 references with the strongest citation bursts. Figure 8A reveals that 20% (5/25) of the citation burstness occurred in 2011, 2012 (3/25,16%), 2014 (3/25,16%) ranked second juxtaposed. The article with the strongest burstness (strength = 140.25) was entitled “Molecular mechanisms of necroptosis: an ordered cellular explosion” (Vandenabeele et al., 2010), a review published in Nature reviews. Molecular cell biology by Peter Vandenabeele et al., in 2010, with citation burstness from 2010 to 2015. Moreover, this article also gets the highest LCS, followed by the production of WANG HY (Wang et al., 2014) and CAI ZY (Cai et al., 2014). We found that among the top 25 references with the strongest citation bursts, “Pasparakis, Manolis, 2015, Nature, V517, *p* 311 (Pasparakis and Vandenabeele, 2015),” (2019–2020, strength 68.21) and “Galluzzi, Lorenzo, 2018, Cell death and differentiation, V25, P486 (Galluzzi et al., 2018)”, (2018–2022, strength = 61.36) were the recent high-citation references, while their LCS was not very high until 2022. Furthermore, it is noteworthy that seven references were still in burstness among the top 50 references (Figure 8A), which implies that the research on necroptosis in cancer will undoubtedly continue to evolve in the coming years.

### 3.6 Keyword co-occurrence, clusters, and evolution

Keywords are at the core of a paper that reflects the current knowledge basis and informs the direction or trends of a related academic field. In order to analyze keyword co-occurrence, we used VOS viewer to present the keyword co-occurrence (Table 5; Supplementary Figure S1A) and cluster analysis (Supplementary Figure S1B). In total, 7,495 keywords were extracted, of which 353 occurred more than ten times, and 32 occurred more than 100 times. We can detect the highest frequency co-occurrence keywords from the density map (Supplementary Figure S1A), illustrating the research hotspot in recent years. As we can see from Figure 10; Table 5, “necroptosis” was the most crucial term with 1,244 co-occurrences, followed by “apoptosis” (*n* = 1,032), “cell-death” (*n* = 561), “activation” (*n* = 395), and “cancer” (*n* = 362).

**TABLE 5:** The top 20 terms of necroptosis in cancer research.

Rank	Keywords	Counts	Rank	Keywords	Counts
1	necroptosis	1,244	11	autophagy	301
2	Apoptosis	1,032	12	NF-κB	285
3	cell-death	561	13	inflammation	264
4	activation	395	14	domain-like protein	227
5	cancer	362	15	TNF	193
6	programmed necrosis	357	16	mechanisms	187
7	necrosis	341	17	inhibition	182
8	MLKL	324	18	oxidative stress	171
9	expression	311	19	kinase	157
10	death	305	20	RIPK3	151

#MLKL, mixed lineage kinase domain-like protein; TNF, tumor necrosis factor; NF-kB, nuclear factor k-light-chain-enhancer of activated B cells; RIPK3, receptor-interacting protein kinase-3.

The network map is clustered in Figure 10A, exhibiting the knowledge structure of necroptosis. We set the “minimum number of occurrences of a keyword” as “50”, and 62 keywords meet the threshold. The keywords were divided into 3 clusters, different colors respectively representing different clusters. Cluster 1 (red) is the most numerous cluster with 32 co-occurrence keywords: necroptosis, apoptosis, autophagy, necrosis, cancer, etc. Each cluster is extraordinarily homogeneous within the keywords based on the link strength of keyword co-occurrence.

Overlay visualization reflected the high-frequency keywords (*n* = 62), which is beneficial to investigate the evolution trajectory and phase attribute of the basic knowledge. Supplementary Figure S1C is the overlay visualization of keywords related to necroptosis and cancer. Since their color was yellow, we can figure out that ferroptosis, pyroptosis, and prognosis are emerging terms.

**FIGURE 9:**
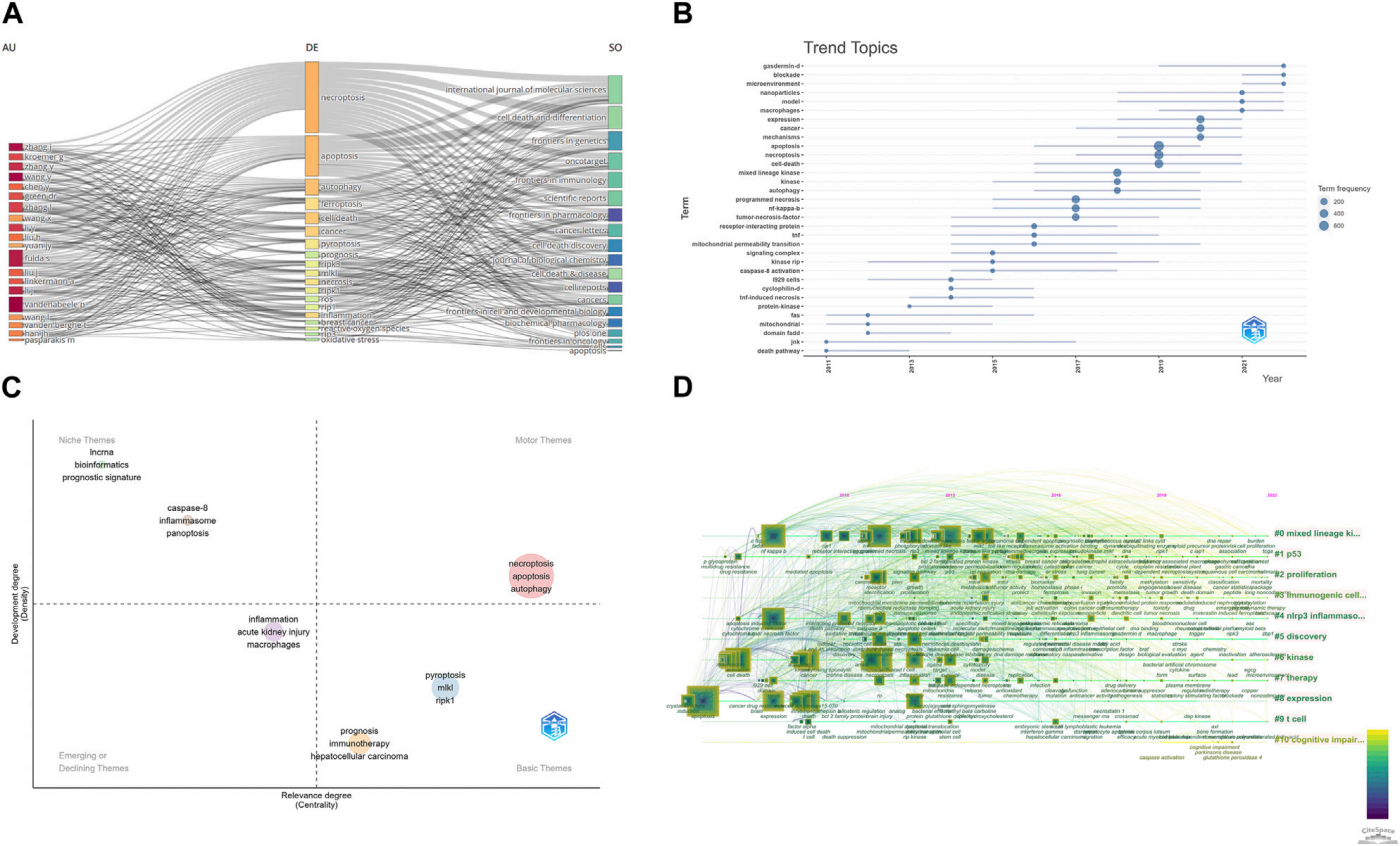
**(A)** Three-Fields Plots of author, journal, and keywords. **(B)** Topic trends from 2006 to 2022. **(C)** Thematic Map in this field. **(D)** A timeline view of keywords related to necroptosis in cancer research.

In CiteSpace, we constructed the timeline view, visually demonstrating the stage hotspots from the time dimension (Figure 9D). As we can see, ten of eleven clusters (except for #10) are still ongoing. And we detected 25 keywords with the most vigorous citation burst (Figure 8B). As shown, 24% (6/25) of the keywords appeared first citation burst in 2012, followed by a juxtaposed 2011 (3/25, 12%). Notably, two keywords, RIPK1 and iron, were in burstness until 2022, which implies that the sustained prosperity of the study related to necroptosis and cancer over extended periods in the future. The keyword with the strongest burstness (strength = 12.46) was “tumor necrosis factor,” with its citation burstness from 2008 to 2014.

We also use “bibliometrix” packages to perform some keyword analysis. Figure 9A shows the relationships between affiliations, authors, and keywords in necroptosis and cancer treatment. Figure 9B shows the trend topics and their prevailing year; we can see that “gasdermin-d,” “blockade,” “microenvironment,” and “nanoparticles” are still hot topics in this field. Figure 9C is the Thematic Map of keywords. The Thematic Map is a two-dimensional diagram constructed using the Density Index as the vertical coordinate and the Centrality Index as the horizontal coordinate. Density represents the strength of connection of the basic knowledge units within a topic. The higher the density of a topic, the higher the maturity of that topic. Centripetalism represents the strength of a topic’s connections to other topics. A higher value of centripetality indicates that an issue is more closely linked to other topics and that it is in a definitive core position. The system is divided into four quadrants based on the density and centroid values: the first quadrant is for motor themes, which refers to highly mature core themes; the second quadrant is for developed and isolated themes, which refers to highly mature isolated themes; the third quadrant is for emerging or disappearing themes, which refers to emerging or disappearing theme; the fourth quadrant is for basic and transversal themes, which refers to low maturity themes that are likely to become research hotspots or trends in the future. And as shown in Figure 9C, “necroptosis,” “apoptosis,” and “autophagy” are all motor themes.

## 4 Discussion

### 4.1 General information

As of 16 September 2022, the most recent updates on the cumulative total of 2,216 English papers related to necroptosis and cancer were published in 685 academic journals by 13,009 authors in 789 institutions from 75 countries/regions. Mutations of annual output and studies trends are critical indicators for the development speed and research concentration in this field (Gao et al., 2019; Qin et al., 2020). The investigation related to necroptosis and cancer was officially initiated in 2006, 1 year after Alexei Degterev first proposed the term “necroptosis” (Degterev et al., 2005), while the production at the beginning was still at a low ebb (Figure 2), which implies that the study of necroptosis is still in its infancy. It can be figured out from Figure 2 that the total number of publications continued to increase from 2010, and it is still ongoing now. Especially from 2010 to 2021, the quantity of literature was at a stable growth stage. During this period, the star pathway consisting of key molecular RIPK1, RIPK3, and its substrate MLKL, which serves a critical function in regulating this novel cell death pathway, was proposed (Sun et al., 2012; Weinlich et al., 2017). RIPK1 is a receptor-interacting serine/threonine-protein kinase, which was known as an essential regulator in apoptosis, redefined necroptosis, and paved the way for the new research area of cell death. Then, between 2018 and 2021, article output related to necroptosis and cancer grew explosively, and the number of publications reached 359 in 2021. In summary, it can be seen that necroptosis in cancer research has attracted mounting researchers’ attention and has a promising development landscape in the future.

Is there any distribution discrepancy among countries/institutions in necroptosis and cancer research? We used R to conduct Figures 3A–C. The results showed that China, the United States, and Germany were the top 3 countries in productivity, which suggests that these three countries are leading contenders in necroptosis and cancer research. Moreover, South Korea, Belgium, Italy, England, France, and Australia were among the top 10 countries that may lead to transformative discoveries. The top 10 institutions were from five countries; three were from China, while the rest were from the United States, Belgium, and France. The University of Ghent, the Chinese Academy of Sciences, and the Flanders Institute for Biotechnology are the top three most productive institutions. Although we can see that the United States has been an active and influential country since 2005, China started later but has become one of the highest-yield contributors in recent years and has played a vital role in the global cooperation network. This result may be associated with these countries’ socioeconomic development and financial prosperity. Besides, close partnerships between different countries/regions, especially the United States, suggested that research related to necroptosis and cancer had attracted significant academic interest worldwide. We can figure out that the United States was the top collaborating center, while China seems to need more scholarly communication compared to the United States as the top-contributed country. As the only developing country in the top 10 productive countries/regions, regardless of the publications or the institutional distribution, it occupies an important position in this field. However, as shown in Figure 4B, Chinese institutions maintain a healthy level of cooperation with each other while lacking communication with foreign academic organizations. Hence, it is strongly encouraged that the institutions from China should break academic obstacles, cooperate, and communicate with other countries to facilitate the development of the necroptosis and cancer field. In recent years, scientific cooperation between countries has suffered a significant blow because of COVID-19, but cooperation is the general trend. This is dictated by the laws inherent in the development of science, and, despite setbacks, the overall trend is irreversible. Understanding inter-state cooperation in specific fields helps us gain insight into disciplinary development characteristics.

**FIGURE 10:**
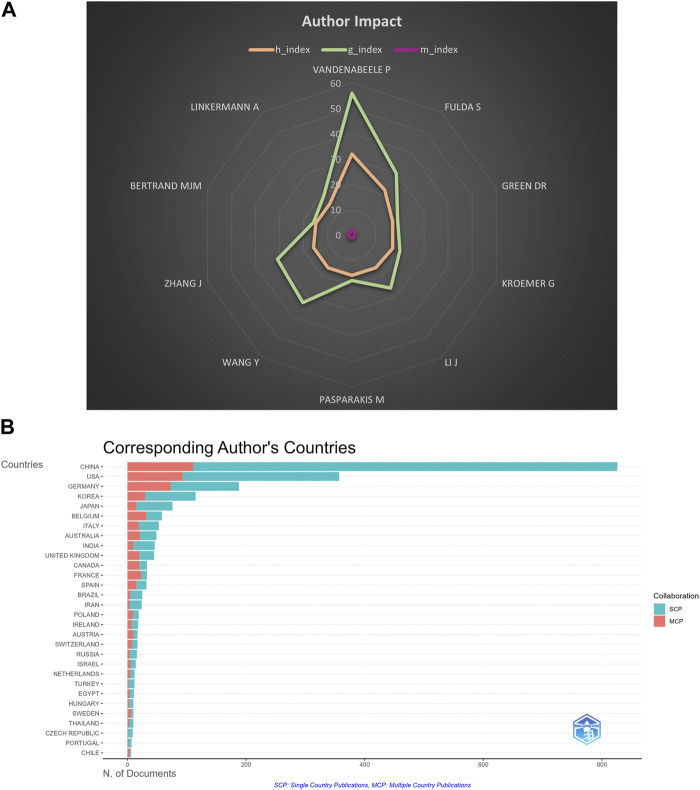
**(A)** H index, G index, and M index of top 10 productive authors. **(B)** Collaboration of the corresponding author.

The scholars who made notable contributions in a specific field, acting as a “weather vane,” may provide numerous directions and guidelines (Kodonas et al., 2021). Hence, it is duty-bound to investigate them in our bibliometric study. At least 13 papers were published by anyone in the top 10 productive authors. Among the top 10 active authors, five were from Europe, three were from China, and the remaining two were from America. This finding may indicate that European researchers are influential in necroptosis and cancer research. In our analysis (Figure 5; Table 2), Vandenabeele, Peterb published the most papers, while Degterev, A had the most co-citations. Meanwhile, we can see that only two scholars are the top 10 active authors and the top 10 co-cited authors, namely, Vandenabeele, Peterb, and Linkermann, Andreas. The finding may imply that these two researchers play a significant role in necroptosis and cancer research. Moreover, the map of co-cited authors reflects influential researchers (Liang et al., 2017). Degterev, A (1,082 co-citations) ranked first, followed by Galluzzi, L (967 co-citations), and He, sd (659 co-citations). Alexei Degterev, a researcher at the Cell Biology Department of Havard Medical School, has been committed to researching cell death for the past 20 years, especially necroptosis. The article he published in 2005, entitled “Chemical inhibitor of non-apoptotic cell death with therapeutic potential for ischemic brain injury.” (Degterev et al., 2005), ranked top 3 of the co-cited references. In this study, Alexei Degterev and his group initially demonstrated the existence of a common non-apoptotic death pathway, “necroptosis,” and identified a specific necroptosis inhibitor, necrostain-1. This study pioneers research related to necroptosis in the human pathologic area. Moreover, In 2008, Alexei Degterev and his group figured out that RIP1 kinase act as the critical upstream kinase of the activation of necroptosis in the article “Identification of RIP1 kinase as a specific cellular target of necrostatins” (Degterev et al., 2008). This paper ranked in the top 25 references with the most vigorous citation bursts (strength = 91.83) from 2008 to 2013. In Figure 10B, we can see that most of the corresponding authors were from a single country, indicating that closer academic-country collaboration is needed.

The H index is a combination of the publication and the citation to measure the impact of a scientist who has published at least H papers that have been cited at least H times, and is widely used to measure the impact of scientific and technical literature or authors (Hirsch, 2005). G index and M index are indicators performed according to the H index. The top ten authors with the most extensive H index were Vandenabeele *p*, Fulda S, Green DR, Kroemer G, Li J, Pasparaki M, Wang Y, Zhang J, Bertran M, and Linkermann A. As shown in Figure 10A; Supplementary Table S1, we can figure out that Vandenabeele *p*, Fulda S, Wang Y, and Zhang J are influential scientists in this field.

Journals and co-cited journals analysis in Table 3 suggested that only 463 papers were published in the top 10 active journals, accounting for only 20.89% of all publications. *Cell Death* and *Disease* (*n* = 106) published the most articles about necroptosis and cancer, followed by *Cell death and differentiation* (*n* = 62), *International journal of molecular sciences* (*n* = 52). Thus, it can be concluded that the studies on the underlying mechanism of necroptosis in the tumor are hotspots over extended periods. As for co-cited journals, *Cell* (*n* = 6,763) is the journal with the highest number of co-citations, followed by *Nature* (*n* = 6,083), *Cell death and differentiation* (*n* = 4,971), implying that these academic journals were focused on necroptosis and cancer research*.* As shown in Table 3, there are five journals cited more than 4,000 times. Meanwhile, journals in the Q1 JCR region with high IF occupied the vast majority of the top 10 journals (70%) and co-cited journals (80%), implying that these academic journals play critical roles in the necroptosis and cancer research field. Therefore, analyzing the distribution of document sources can be of great help in discovering core journals in the necroptosis-related research field. And the results showed that the cited articles are primarily from high-influential journals, which indicated that the research on necroptosis and cancer would attract more and more academic attention.

The analysis of co-cited references could broadly reflect the knowledge base in this research field (Chen et al., 2006; Chen, 2017). The top 10 highly co-cited references correlated with the necroptosis molecular mechanism. Furthermore, after comprehensively collecting the data of authors, co-cited authors, and co-cited references, we can see that professor Peter Vandenabeele was the only scholar who occurred in all three groups, indicating that he is an authoritative scholar in this field. His group would be considered excellent potential collaborators in the necroptosis and cancer research area. Factorial analysis performed according to references is shown in Supplementary Figure S2.

### 4.2 The hotspots and frontiers

In bibliometrics, keywords/terms co-occurrence (Figure 8; Table 5) can provide the readers with a comprehensive understanding of research themes and core contents of an academic field, and the timeline view (Figure 9A) can reflect the evolution of hotspots in the corresponding field. As is shown in Table 5, the keywords with high occurrence frequency are necroptosis (*n* = 1,244), apoptosis (*n* = 1,032), cell death (*n* = 561), activation (*n* = 395), necrosis (*n* = 584), cancer (*n* = 362), programmed necrosis (*n* = 357), etc. In addition, according to the cluster analysis conducted in Figure 8, three-color clusters were formed, which reflects the research hotspots and development frontiers related to necroptosis and cancer. In this section, we aim to illustrate the main contents and knowledge structure in the field of necroptosis.

#### 4.2.1 Pathway mechanism

Cell death can be divided into programmed cell death (PCD) and non-programmed death. PCD, an intracellular death program executed by well-defined signal transduction mechanisms, takes many forms, including apoptosis, pyroptosis, necroptosis, and ferroptosis. Nevertheless, non-programmed death refers to necrosis. All forms of cell death play a prominent role in homeostasis maintenance and disease development.

Apoptosis, pyroptosis, necroptosis, and ferroptosis are strictly regulated procedures, while differences exist, such as morphological phenotypes, triggers, effector proteins, and released substances. The executed protein location changes play a vital role in the process of cell death. For instance, the translocation of caspase-3 from the cytoplasm into the nucleus is a crucial step in promoting the apoptosis process, which can regulate DNA replication and protein synthesis by affecting multiple macromolecular biosynthesis pathways (Kopeina et al., 2018). As a result, nuclear condensation, DNA fragmentation, and nuclear rupture can occur (Wang et al., 2017). During pyroptosis, the N-terminal fragment of gasdermins has the ability to localize and form cores within the cell membrane. The damaged cellular organelles and DAMPs will be released to the extracellular space non-selectively (Ding et al., 2016; de Vasconcelos et al., 2019). Lysosomal and mitochondrial damage appear before the membrane rupture, which may be related to NLPR3 (Zhou et al., 2011). Like gasdermins, MLKL can also lead to cell membrane rupture. That, previous studies have shown that phosphorylated MLKL can also translocate into lysosomes, mitochondria, endoplasmic reticulum, and nucleus, and these processes occur after MLKL-membrane binding (Wang et al., 2014; Yoon et al., 2016). Activated RIPK3 and MLKL shuttle continuously between the cell membrane and nucleus may help promote necrosome formation (Weber et al., 2018). Nevertheless, whether the translocation of MLKL affects organelle function and further affects cell death requires further study. The primary manifestation of ferroptosis is significant changes in mitochondrial morphology, which is an important indicator to determine whether a cell lives or dies. Ferrous iron can induce oxidative damage in the mitochondrial inner membrane because the inner membrane is particularly susceptible to oxidative damage and contains a rich source of unsaturated fatty acids compared to the outer membrane (Bogacz and Krauth-Siegel, 2018).

Necroptosis, also known as programmed necroptosis, is a pattern of regulated cell death that simulates some of the characteristics of necrosis and apoptosis. The underlying mechanism was initially discovered since self-destructive behavior also exists when apoptosis is blocked. The execution of necroptosis pathways does not depend on caspase activity but depends on the RIPK3-dependent phosphorylation of MLKL.

Several extrinsic and intrinsic stimuli have been shown to induce the necroptosis pathway. In general, when the receptors including tumor necrosis factor receptor 1 (TNFR1), interferon receptor (IFNR), Toll-like receptor 3/4/9 (TLR3/4/9), and DNA-dependent activator of IFN regulatory factors (DAI) are activated, they can trigger the activation the RIPK kinase family, and further, initiate necroptosis (Takemura et al., 2015; Kesarwani et al., 2016; Brault et al., 2018; Huang et al., 2018; Lee et al., 2019).

The most predominant mechanism of necroptosis regulation is death receptors-participated-signaling pathways, which are closely related to apoptosis. The most classic example is the tumor necrosis factor-α (TNF-α) induced pathway. When TNF-α binds to the TNFR1 on the cell membrane surface, the C-terminal death domain of TNFR1 drives the process by recruiting TNF receptor 1-associated death domain protein (TRADD), RIPK1, Tumor necrosis factor receptor-related factor (TRAF2) and cellular inhibitors of apoptosis 1 and 2 (cIAP1/2), and further recruited linear ubiquitin Chain assembly complex (LUBAC) (Galluzzi et al., 2017). These binding events form scaffolding protein complexes, which serve as a platform to create the TNFR complex I (Pasparakis and Vandenabeele, 2015; Galluzzi et al., 2017). Complex I can activate the NF-κB signaling pathway, thereby promoting cell survival and inducing inflammatory responses, which is relevant in the poly-ubiquitination of RIPK1 (Hayden and Ghosh, 2014). The consequence of RIPK1 polyubiquitination is mainly induced by cIAP1/2 and LUBAC (Dondelinger et al., 2016). On the one hand, cIAP1/2 can undergo RIPK1 ubiquitin modification owing to the E3 ubiquitin ligase of RIPK1. Through this ubiquitin modification, RIPK1 can further recruit transforming growth factor-beta active kinase 1 (TAK1) and TAK1 binding protein (TAB) (Newton et al., 2008; Dynek et al., 2010; Draber et al., 2015), which in turn activates the NF-κB signaling pathway (Witt and Vucic, 2017; Annibaldi and Meier, 2018). On the other hand, LUBAC is capable of inducing the formation of M1-linked ubiquitin chains, thereby promoting the recruitment of nuclear factor-κB essential modulators (NEMO) on complex I (Hadian et al., 2011). NEMO is a regulatory subunit of the IκB kinase (IKK) complex, containing IKK1/IKKα and IKK2/IKKβ (Ting and Bertrand, 2016). Moreover, cIAP1/2 and LUBAC also assist the interaction between TANK binding kinase 1 (TBK1)/IKKε and complex I, which is vital for TNF-induced cell death (Lafont et al., 2018). In summary, the recruitment of these molecules on complex I will further activate the NF-κB pathway.

Complex I is considered a critical checkpoint of cell survival since activating the NF-κB pathway will induce the expression of pro-survival genes. For instance, cIAP2 expression degrades ubiquitinated IκB, an inhibitory protein of NF-κB, thereby releasing related molecules into the nucleus and promoting cell survival (Annibaldi and Meier, 2018). As described above, ubiquitination of various components in complex I stabilize complex I on cell membranes and inhibit complex II formations. However, when these components are inhibited, they will be released from cell membranes and form complex II. Through CIAPs inhibition or deubiquitination of RIPK by deubiquitinating enzymes cylindromatosis (CYLD), complex I recruit FADD, RIPK1, and caspase-8 and forms Complex II. There are two forms of complex II: TRADD-dependent (complex II a) and RIPK1-dependent (complex II b). RIPK kinase activity can distinguish these two mechanisms when inducing cell death (Kreuz et al., 2001; Blackwell et al., 2013; Ting and Bertrand, 2016). Regardless of the forms of TNFR complex II, as long as the caspase-8 activity is not inhibited, the outcome of such signal transduction is apoptosis. Once inhibiting the catalytic activity of caspase-8, the inhibitory effects of caspase-8 on RIPK1 will be relieved so that RIPK1 can phosphorylate RIPK3 and recruit MLKL, forming the necrosome (Mompeán et al., 2018; Newton et al., 2019). Under the regulation of inositol phosphate (IP), phosphorylated RIPK3 could also lead to phosphorylation and oligomerization of MLKL (Dovey et al., 2018). After that, MLKL intercalates into the cell membrane since MLKL is capable of binding to the lipids having a negative change (Dondelinger et al., 2014; Quarato et al., 2016), which pumps magnesium ions out and pumps calcium ions in, leading to the intracellular accumulation of calcium (Cai et al., 2014). Therefore, cell membrane permeability increases, and cellular damage-associated molecular patterns (DAMPs) in the cytoplasm will be relieved (Kaczmarek et al., 2013; Wang et al., 2014), such as IL-1a, IL-33, and HMGB1, resulting in tissue inflammation and organ damage. What’s more, MLKL will activate the large GTPase dynamin-related protein 1 (Drp1) in mitochondria through phosphoglycerate mutase 5 (PGAM5), accompanying this is an elevated accumulation of ROS and mitochondrial division (Remijsen et al., 2014; Park et al., 2018; Li et al., 2022).

Except for TNF-α, TNF-related apoptosis-inducing ligand receptor 1/2 (TRAILR1/TRAILR2), death receptor 3 (DR3), death receptor 6 (DR6), Fas cell surface death receptor (FAS/CD95), LPS and even members of interferon (IFN) family have the ability to trigger the necroptosis, while in different signaling pathways (Tsukamoto et al., 2010; McComb et al., 2014; Cekay et al., 2017; Nahacka et al., 2018; Tsukamoto et al., 2018). In summary, several triggers and pathways are working for the precise execution of necroptosis; one thing that is not in dispute is that the process of necroptosis depends mainly on the RIPK1-RIPK3-MLKL necrosome.

#### 4.2.2 Role of necroptosis in tumor

It is well-known that tumor development is closely correlated with cell death, and necroptosis makes no exception. Existing evidence suggests that necroptosis can promote and inhibit tumor development, depending on tumor subtype, tumor stage, and tumor grade (Qin et al., 2019). Most tumor cells show resistance to necroptosis thanks to the low expression of RIPK3, and the tumor would flourish. Researchers have observed that the expression of RIPK3 was downregulated in human tumor samples, including Acute Myeloid leukemia (AML) (Nugues et al., 2014), Chronic lymphocytic leukemia (CLL) (Höckendorf et al., 2016), colorectal cancer (Bozec et al., 2016), and breast cancer (Koo et al., 2015). On the one hand, disruption of necroptosis in tumor cells would lead to tumor development. Several necroptosis-related molecules were downregulated in tumor cells, such as CYLD. CYLD is a type of deubiquitinating enzyme facilitating necroptosis, which plays a vital role in the process of necroptosis. Liu et al. (2012) confirmed that CYLD was suppressed in CLL. Moreover, during necroptosis, the release of DAMPs from cells was considered inflammatory cell death, so the NF-κB and MAPK pathways would be activated, promoting tumor invasion and metastasis. It was reported that necroptosis could induce tumor cells to release inflammatory factors SAP130 in pancreatic ductal adenocarcinoma (PDA) so that the receptor could recognize it and induce immunosuppressive tumor microenvironment, and facilitate the progression of PDA (Seifert et al., 2016). The necroptosis-induced tumor microenvironment can promote tumor invasion and metastasis and determine tumor lineage subtype. Marco Seehawer et al. (2018) found that the necroptosis tumor microenvironment could cause hepatocellular carcinoma (HCC) changes into intrahepatic cholangiocarcinoma (ICC) in a mouse model of HCC. On the other hand, the intervention of cells in the tumor microenvironment could also facilitate tumor development. Recent studies have shown that the extravasation of tumor cells across the endothelium was crucial in tumor metastasis. The tumor cells’ extravasation and metastasis were facilitated by activating death-receptor 6 (DR6) induced necroptosis in endothelial cells (Strilic et al., 2016). While Utilizing Nec-1 to treat mice or specific knockout RIPK1/MLKL could inhibit the necroptosis in endothelial cells, further decreasing tumor extravasation. Therefore, inhibiting the necroptosis of endothelial cells may be a novel strategy for cancer treatment. In summary, these studies revealed the role of necroptosis in tumor growth and metastasis and further lay a theoretical foundation for targeting necroptosis to inhibit tumor development.

Simultaneously, several clinical and pathological studies have discovered that necroptosis may play an essential role in eliciting immunogenic cancer cell death and promoting innate immune surveillance functions. For instance, tumor cells could release IL-1α during the necroptosis process to activate dendritic cells (DCs). The activated DC cells can produce IL-12 and activate CD8^+^ T cells to induce an anti-tumor immune response (Schmidt et al., 2015; Takemura et al., 2015). At the same time, Nader Yatim et al. found that DAMPs from necroptotic tumor cells would strongly lead to the elevated expression of CD8^+^ T (Yatim et al., 2015). Moreover, Young Jun Kang et al. confirmed that NKT cells took part in RIPK3-induced anti-tumor immunogenic response, and the deletion of RIPK3 may destroy the process of NKT cell activation (Kang et al., 2015). As for specific cancer, some crucial molecules in the necroptosis process have been confirmed to correlate with cancer patients’ survival. Ling He and his group have figured out that low expression of MLKL was significantly associated with a shortened disease-free survival and overall survival in primary ovarian cancer patients (He et al., 2013). Furthermore, high MLKL expression was negatively correlated with histological grade and lymphatic metastasis, which indicates MLKL-induced necroptosis may be the target of cancer treatment (Ruan et al., 2015). Overall, necroptosis is a double-edged sword in tumor development; more research is needed to explore these mechanisms in the different tumor microenvironments.

#### 4.2.3 Application of necroptosis in cancer treatment

**FIGURE 11:**
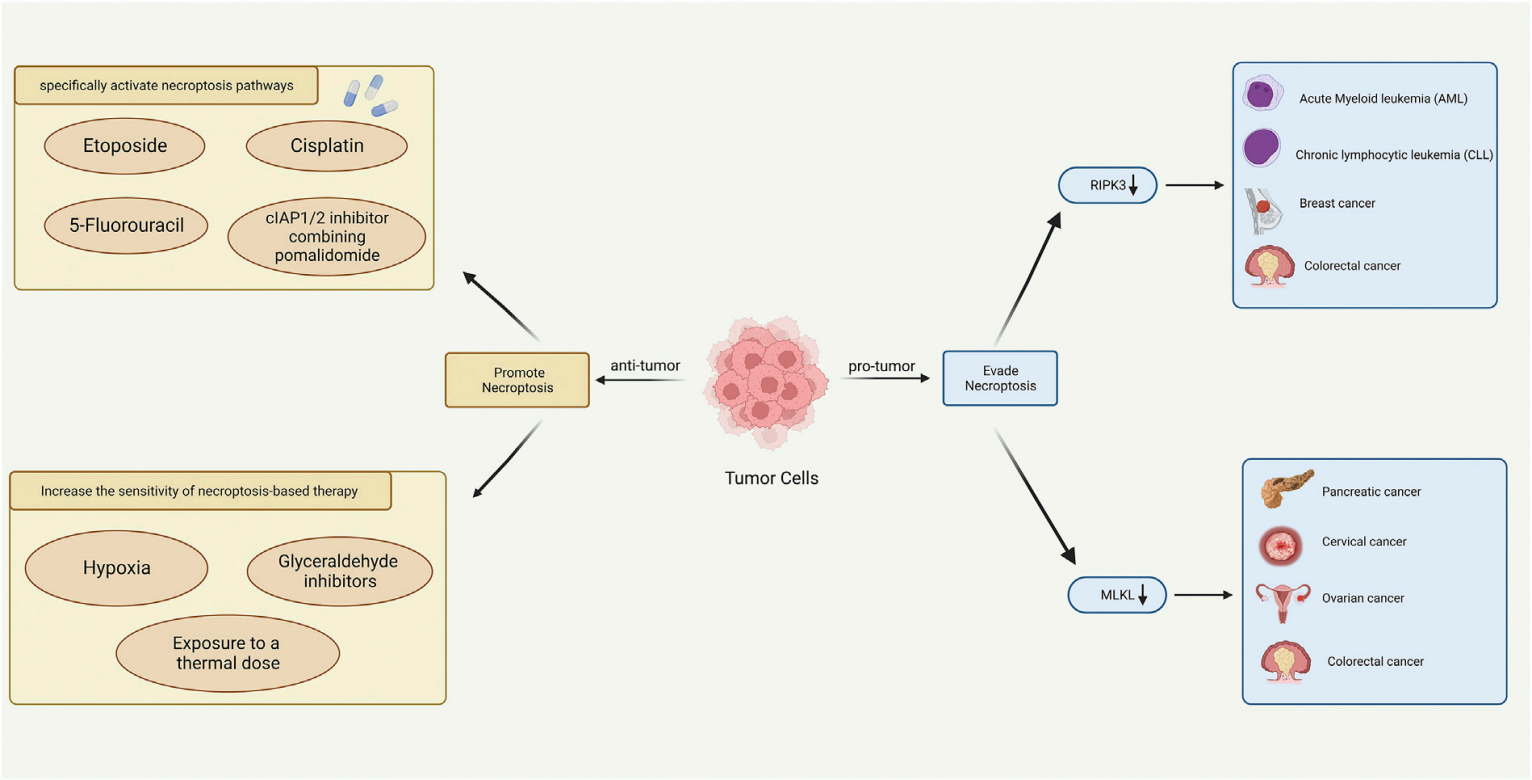
Role of necroptosis in tumor progression (right) and cancer treatment (left) (Created with BioRender.com).

Although most anti-tumor drugs in clinical use can contribute to the induction of apoptosis (Fulda, 2015), chemotherapy resistance and apoptosis resistance are also the biggest challenges in cancer treatment. First, it is readily conceivable that activating necroptosis pathways in tumor cells could largely overcome resistance to induced-apoptosis drugs (Gong et al., 2019). Several studies had shown that when Caspase activity was inhibited, some chemotherapy drugs could induce necroptosis in tumor cells, including etoposide, 5-fluorouracil, and cisplatin (Tenev et al., 2011; Brown et al., 2015; Seifert et al., 2016). Yuanqin Yang et al. has confirmed that activating the ZBP1-MLKL necroptotic signal pathway could regulate the release of mitochondrial DNA and further enhance antitumor immunity, providing novel treatment insights in overcoming radiotherapy resistance (Yang et al., 2021). Simone Fulda and his group found that cIAP1/2 inhibitor combining pomalidomide could induce necroptosis in drug-resistant tumor cells during ALL treatments (Rohde et al., 2017). Moreover, exchanging physiochemical factors may contribute to sensitizing tumor cells to necroptosis-based therapy. Tumor cells may undergo classic necroptotic events under hypoxic conditions by either inhibiting RIPK1 and RIPK3 expression or reprogramming glycolytic metabolism (Huang et al., 2013). Mouratidis et al. (2015) demonstrated that RIPK3 protein levels of human colon cancer cells were increased after thermal exposure. This suggests that heat therapy and drugs targeting hypoxia may synergize with necroptosis as an applicable therapy for future cancer treatment. We concluded the role of necroptosis in tumor progression and cancer treatment in Figure 11.

Therefore, it is an integrated approach to overcome chemotherapy resistance via inducing necroptosis in tumor cells, which may accompany novel problems in clinical application. How to target tumor cells and not disrupt the biological function of normal cells may be a core issue that remains to be addressed.

## 5 Strengths and limitations

The current study is the first bibliometric study to explore the emerging topic and provide a comprehensive knowledge map related to necroptosis and cancer. Moreover, compared to the traditional reviews, our results show more intuitive evidence on research foci and trends in multiple dimensions. Inevitably, some limitations exist in our study. Firstly, we only retrieved the data from the WoSCC database, and we only included the original articles and reviews written in English. That way, a few studies should have been included. Secondly, all data were collected by CiteSpace and VOSviewer based on machine learning; some details must be ignored since they cannot distinguish synonyms, antonyms, and abbreviations. Thirdly, the papers included in the WoSCC database constantly changed. Some newly published high-quality articles may be ignored because co-cited counts need to be higher, and the uneven quality of the collected data also reduces the credibility of bibliometric analysis. Overall, our bibliometric research still lays a solid foundation for scholars who will work on necroptosis and cancer.

## 6 Conclusion

In conclusion, research focusing on necroptosis and cancer has entered into a vigorous development period due to various high-quality publications. According to the search results, our article is the first bibliometric analysis using CiteSpace and VOSviewer to identify the research trends and hotspots of necroptosis in cancer. Active cooperation exists between several institutions worldwide, of which the United States is the crucial collaborating bridge. The molecules in necroptosis pathways, like RIPK1/RIPK3/MLKL, were still one of the most appealing stars in this field and will be a significant focus in the future. Thus, understanding the necroptosis accounts in cancer treatment by evaluating the efficacy of necroptosis-related drugs are urgent questions that need to be addressed urgently and also the future directions of necroptosis research for cancer diagnosis and treatment. We believe the results of our study would lay a solid foundation for further research about necroptosis in cancer.

## Figures and Tables

**Table:** 

**RIKP**	Receptor-interacting serine/threonine protein kinase
**MLKL**	Mixed lineage kinase domain-like pseudokinase
**TNF**	Tumor necrosis factor receptor
**TLR**	Toll-like receptors
**TAP-MS**	Tandem affinity purification coupled with mass spectrometry
**RNA-seq**	RNA-sequencing
**DCs**	Dendritic cells
**NF-κB**	Nuclear factor-κB
**IF**	Impact factor
**PCD**	Programmed cell death
**TNFR**	Tumor necrosis factor receptor
**IFNR**	Interferon receptor
**TLR**	Toll-like receptor
**DAI**	DNA-dependent activator of IFN regulatory factors
**TRADD**	Death domain protein
**TRAF2**	Tumor necrosis factor receptor-related factor
**cIAP1/2**	Cellular inhibitors of apoptosis 1 and 2
**LUBAC**	Linear ubiquitin Chain assembly comple
**TAK1**	Transforming growth factor-beta active kinase
**TAB**	TAK1 binding protein
**NEMO**	Nuclear factor-κB essential modulators
**IKK**	IκB kinase
**TBK1**	TANK binding kinase
**DAMPs**	Cellular damage-associated molecular patterns
**Drp1**	The large GTPase dynamin-related protein 1
**PGAM5**	Phosphoglycerate mutase 5
**TRAILR**	TNF-related apoptosis-inducing ligand receptor
**DR**	Death receptor
**CYLD**	Cylindromatosis
**FAS**	Fas cell surface death receptor
**IFN**	Interferon
**CLL**	Chronic lymphocytic leukemia
**PDA**	Pancreatic ductal adeno-carcinoma
**HCC**	Hepatocellular carcinoma
**ICC**	Intrahepatic cholangiocarcinoma
**LCS**	Local citation score
**GCS**	Global citation score

## Data Availability

The original contributions presented in the study are included in the article/Supplementary Material, further inquiries can be directed to the corresponding author.
