# Supplementary figures and images for: Global publication trends and research trends of necroptosis application in tumor: A bibliometric analysis
Source: Front Pharmacol. 2023 Apr 24;14:1112484. doi: 10.3389/fphar.2023.1112484 (PMC10164947; doi:10.3389/fphar.2023.1112484)

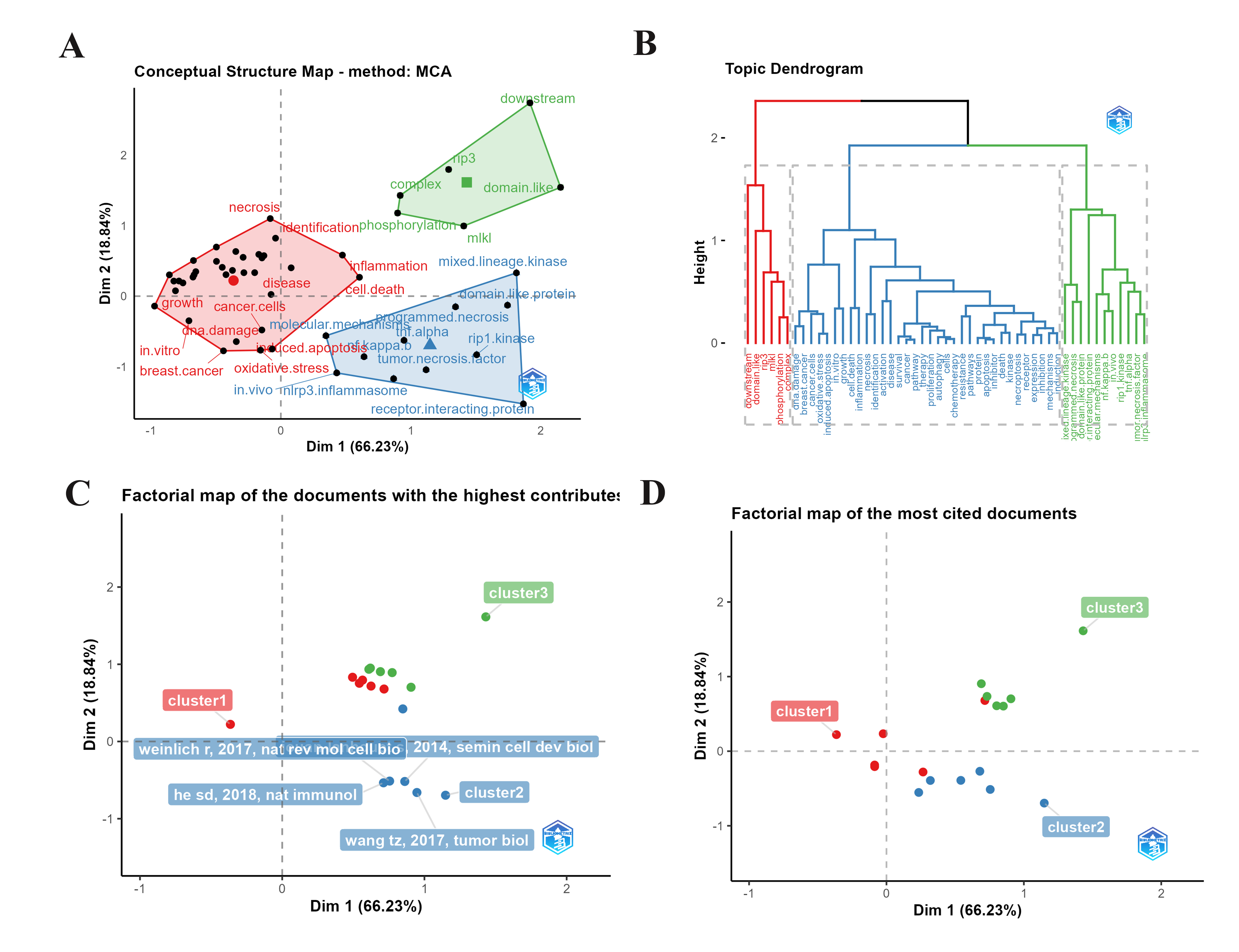

Supplement: Supplementary file 1 [file Image2.TIF]

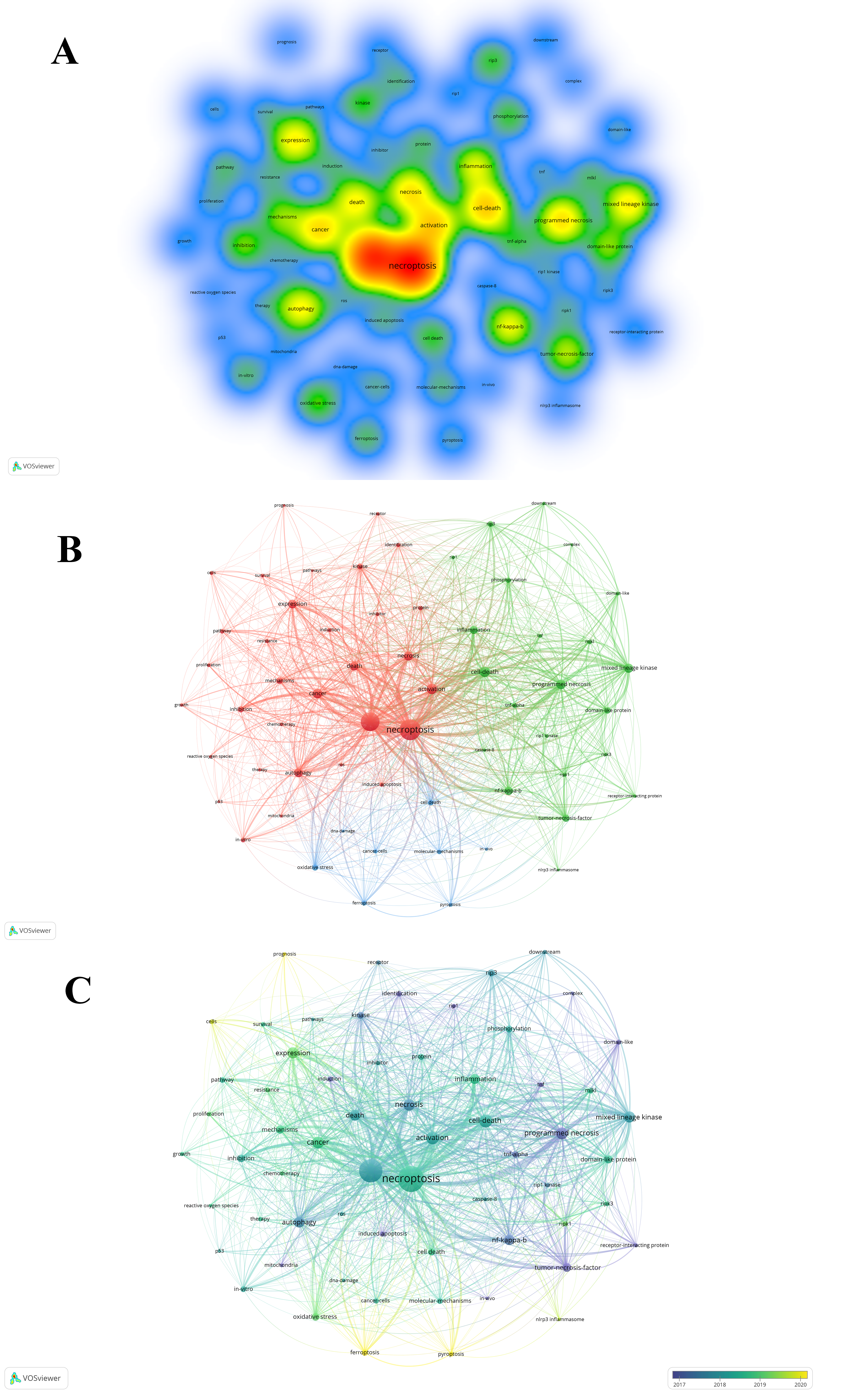

Supplement: Supplementary file 2 [file Image1.TIF]
